# 3D nanofabricated soft microrobots with super-compliant picoforce springs as onboard sensors and actuators

**DOI:** 10.1038/s41565-023-01567-0

**Published:** 2024-01-03

**Authors:** Haifeng Xu, Song Wu, Yuan Liu, Xiaopu Wang, Artem K. Efremov, Lei Wang, John S. McCaskill, Mariana Medina-Sánchez, Oliver G. Schmidt

**Affiliations:** 1 Shenzhen Institute of Advanced Technology (SIAT), Chinese Academy of Sciences, Shenzhen, China; 2 Leibniz Institute for Solid State and Materials Research Dresden (Leibniz IFW Dresden), Dresden, Germany; 3 Shenzhen Institute of Artificial Intelligence and Robotics for Society, Shenzhen, China; 4 Shenzhen Bay Laboratory, Shenzhen, China; 5 Research Center for Materials, Architectures and Integration of Nanomembranes (MAIN), Chemnitz University of Technology, Chemnitz, Germany; 6 Chair of Micro- and NanoSystems, Center for Molecular Bioengineering (B CUBE), Dresden University of Technology, Dresden, Germany

**Keywords:** Mechanical engineering, Biosensors, Materials for devices, Design, synthesis and processing

## Abstract

Microscale organisms and specialized motile cells use protein-based spring-like responsive structures to sense, grasp and move. Rendering this biomechanical transduction functionality in an artificial micromachine for applications in single-cell manipulations is challenging due to the need for a bio-applicable nanoscale spring system with a large and programmable strain response to piconewton-scale forces. Here we present three-dimensional nanofabrication and monolithic integration, based on an acrylic elastomer photoresist, of a magnetic spring system with quantifiable compliance sensitive to 0.5 pN, constructed with customized elasticity and magnetization distributions at the nanoscale. We demonstrate the effective design programmability of these ‘picospring’ ensembles as energy transduction mechanisms for the integrated construction of customized soft micromachines, with onboard sensing and actuation functions at the single-cell scale for microrobotic grasping and locomotion. The integration of active soft springs into three-dimensional nanofabrication offers an avenue to create biocompatible soft microrobots for non-disruptive interactions with biological entities.

## Abstract (web summary)

Here, 3D nanofabrication and elasticity programming of monolithic soft microrobots equipped with magnetic springs with strain response at piconewton forces capable of deformation on micrometre length scales is demonstrated for applications in cell force sensing, cell manipulation and soft actuation.

## Main

Spring components have an important role in energy harvesting and release for microscale organisms in nature. For example, bacteria, sperm or suctoria use their elastic pili, flagella or tentacles for sensing piconewton-scale forces^1^, propulsion^2^ or gripping prey^3^, respectively. Mimicking such complex functions is difficult for artificial micromachines due to the lack of an applicable small-scale spring system. Miniature springs, operative in biological conditions, are challenging to fabricate but essential for soft micromachines to perform biomedical tasks, such as assessing tissue and cell biomechanics, and carrying and releasing cells or therapeutic cargoes^4^. Considering the weak forces that cells can tolerate and generate, spring-based micromachines interacting with them must be stable in aqueous solutions^5,6^ and have adjustable compliance sensitive to down to piconewton-scale forces^7^, with low Young’s modulus and high yield strain. Incorporating springs into such customized micromachines faces the challenge of high-resolution integrated manufacturing of rigid and flexible components with different elasticities.

Previously, micromachines were developed with nanomembranes^8^, bimolecular chains^9^ or natural cells^10^ as their spring components for soft actuation and stem cells delivery. Shape-morphing abilities such as bending and folding based on soft actuators were also realized by linked micro- or nanomagnetic objects^8,11^. Specifically, previous studies have demonstrated the achievement of magnetic soft micromachines by connecting Janus magnetic microbeads with soft linkages^11^. However, the utilization of such prefabricated magnetic beads and their placement on a two-dimensional surface inherently limit their potential for achieving higher levels of structural intricacy and complete three-dimensional (3D) configurations. Unlike their mesoscopic counterparts^12–14^, previous soft micromachines generally had limited structural and mechanical complexity, in particular when fabricated through methods such as self-assembly^9^, layer-by*-*layer deposition^15^, e-beam lithography^8^, micromoulding^16^ and overlay photolithography^17^. In contrast, two-photon lithography (TPL) has enabled the 3D nanofabrication of complex micromachines^18^. However, their applications are limited with conventional materials (for example, IP-DIP photoresist, acrylate derivatives), owing to their low compliance, in the range of micrometres and micronewtons^19^, or their dependency on organic solvents^20^. Hydrogels are promising materials with a low elastic modulus. Nevertheless, the shape morphing of the previously reported hydrogel-based micromachines always relied on stimuli such as intense light^21,22^, temperature change^23^, pH gradients^24,25^ or specific molecules^26^, which limit their applications in enclosed and delicate biological environments—for example, such stimuli are difficult to induce remotely deep within the body for medical applications. Contrastingly, magnetic fields have been widely reported as high-penetration energy sources for micromachines, making remote control feasible^8,9,11^. Complex biomedical tasks require the soft magnetic micromachines to have task-oriented configurations with customized and nanoscale patterned mechanical properties, such as tunable elasticity and sensitivity to very low forces in biologically relevant fluids. To manufacture spring-based soft micromachines using the magnetic field as the single energy source, magnetic-elastic materials compatible with 3D nanofabrication are needed.

**Fig. 1:**
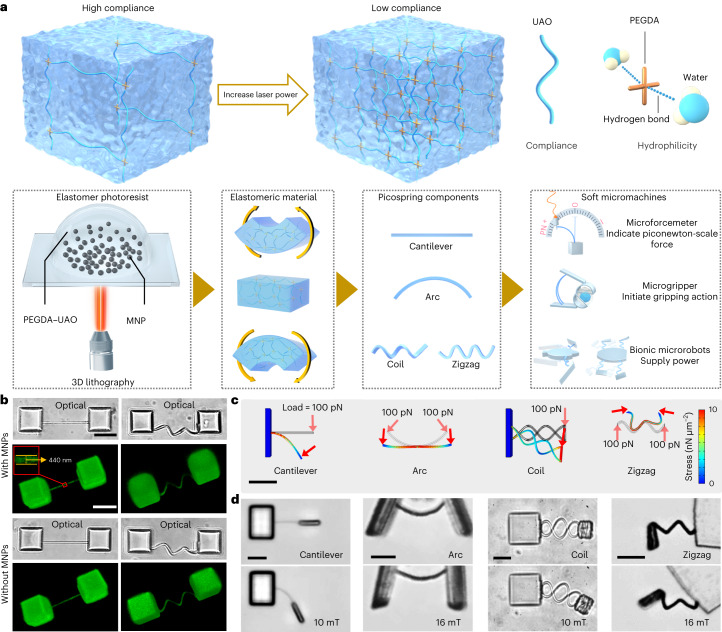
Fabrication of picospring-based micromachines with programmable elasticity distributions. **a**, Schematic illustration of the 3D nanofabrication based on TPL. The photoresist contains an elastomer (urethane acrylate oligomer, UAO), a hydrogel (poly(ethylene glycol) diacrylate, PEGDA) and embedded MNPs. MNPs comprise only 3% of the photoresist to avoid the laser scattering during lithography. The local elasticity is dependent on the spatially programmed fabrication laser power. The picosprings are responsive to piconewton-scale forces, such as those arising from microswimmers or from driving magnetic fields. Picosprings perform specific functions in customized soft micromachines with different configurations. **b**, 3D-reconstructed geometries of the fixed cantilever and coil picosprings based on stacked fluorescence images taken by confocal laser scanning microscopy showing the independence of fabrication geometry on MNP content. Inset: the cantilever width averaged at 440 nm. **c**, Mechanical simulation results showing the deformations of four types of picospring. The load forces are applied parallel to the cross-section of the springs. **d**, Fabricated picosprings (top) and their deformations (bottom) under magnetic loads. Scale bar, 10 μm.

**Extended Data Fig. 1:**
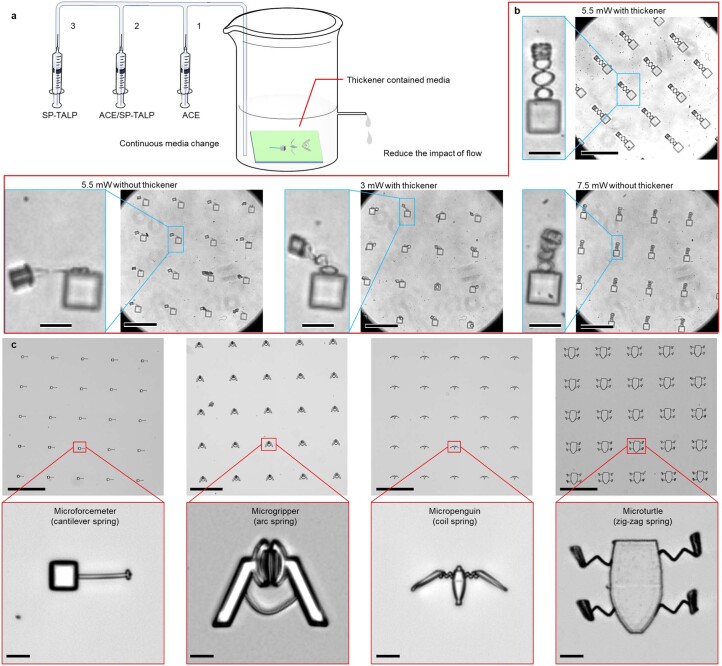
Structural stability of the picospring system during development and media change. (**a**) Schematic illustration of the media changing process with intermediate media containing acetone (ACE) and the sperm cell media (SP-TALP) to avoid damage to the structure when moved from ACE to aqueous solutions. (**b**) Optical images showing the microstructure quality relevant to the laser power and the use of the thickener during media change. Scale bars: 10 μm in the panel of single microstructures and 50 μm in the array images. (**c**) Optical images of four soft micromachines with different types of springs. Scale bars: 100 μm for the array in the upper panels and 10 μm for the single micromachine in the lower panels.

In this Article, we introduce a super-compliant nanostructured spring system with piconewton-scale force sensitivity (picospring) integratable by 3D nanofabrication into functional soft micromachines at the single-cell scale. The picospring components are fabricated by the photo-crosslinking of an elastomer photoresist complex (Fig. 1a). The photoresist contains an elastomer to provide the elasticity, a hydrogel to provide the biocompatibility and hydrogen-bond-based affinity with aqueous solution^27^, and embedded superparamagnetic nanoparticles (MNPs) at about 20 nm to provide magnetization^20^. After the sequential process of exposure, development and media exchange, the picosprings retain their structural integrity and can safely operate in biological solutions (Extended Data Fig. 1; see details in Methods). By locally and monolithically tuning the elasticity of the structural material, customized picospring-based micromachines with biocompatible functions are presented, such as measuring real-time cell forces in situ (microforcemeter), manipulating cells with a protective bucket (microgripper) and performing multimodal locomotion by shape morphing (micropenguin and microturtle). In such micromachines, the picosprings can not only be used individually but also be integrated as a set of cooperative components to enhance the programmability of the soft micromachines.

## 3D nanofabrication of picospring-based micromachines

**Extended Data Fig. 2:**
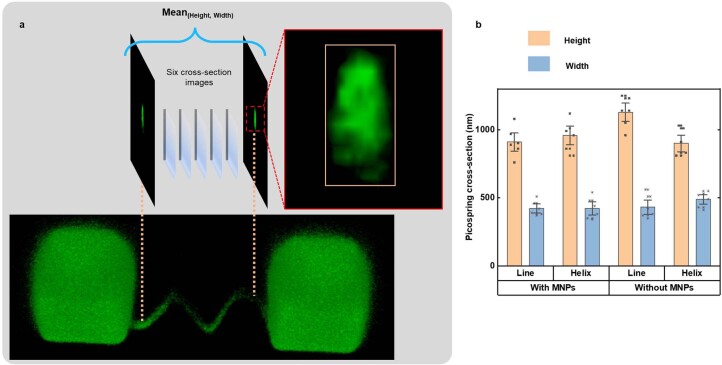
Geometry characterization of the cross-section of the picosprings based on high-resolution confocal laser scanning microscopy. (**a**) Cross-section images using a helix picospring as an example. The average height and width of the picospring of each sample are calculated based on six evenly distributed cross-section images in the 3D-reconstructed image of the picospring. (**b**) Cross-section dimensions of picosprings. The geometry and the embedding of MNPs show no significant influence on the picospring dimensions. Dot plots represent 6 individual images for each group. Bars represent mean±s.d. Source data

Figure 1b shows high-resolution 3D-reconstructed images of the fabricated picospring examples. Picosprings with and without MNPs show no difference in geometric dimensions, with a cross-section of 980 nm × 440 nm (Extended Data Fig. 2). During energy conversion, high compliance leads to large recoverable deformations of the picosprings under ultralow loads. Hence, the stored energy can be visually displayed by the picospring deflection under microscopy and directly utilized for controlling microrobot actions by programming the energy-release timing. Picospring components with various geometries, including cantilevers, arcs, coils and zigzags, can be nanofabricated in three dimensions to meet the compliance and complexity requirements of different soft micromachines for sensing and applying piconewton-scale forces (Fig. 1c,d).

## Elasticity programming in 3D-nanofabricated micromachines

**Fig. 2:**
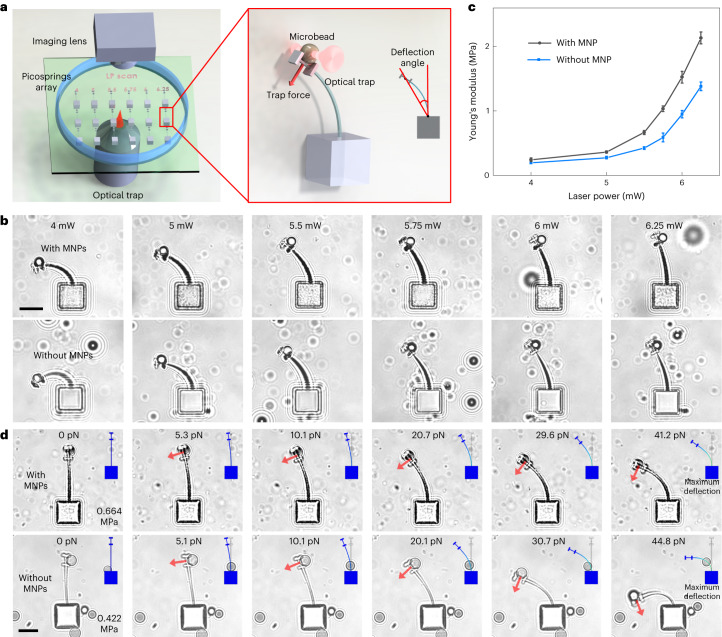
Mechanical characterization of the picospring material based on a cantilever method. **a**, Schematic illustration of the characterization on a picospring array by an optical trap. The characterization structure has a cantilever picospring and two short bars at the free end, forming a holder for an action microbead pulled by the optical trap to stably deform the picospring. The load is applied parallel to the cross-section of the cantilever by the trapped microbead. When the microbead is moved at a negligible velocity to deform the cantilever picospring, the elastic force of the cantilever picospring approximately equals the trapping force provided by the optical trap. Rigid parts are coloured grey and flexible picosprings are coloured blue in the right panel. We use 25 mW to fabricate all nominally rigid parts. **b**, Cantilever picospring deformations under a certain load of 25.4 pN showing a negative correlation between the cantilever deflection and the fabrication laser power. Samples with or without MNPs represent the microstructures based on elastomeric materials with or without MNPs. **c**, Increasing Young’s modulus of the elastomeric material with respect to the fabrication laser power. *n* = 3 independent samples, mean ± s.d. Under small deformations, Young’s modulus *E* of the cantilever picospring is calculated by an approximate formula *E* = *FL*^2^/*3Iθ* (*θ* < 20°), where *F*, *L* and *I* represent the load force, the cantilever length and the moment of inertia of the cantilever, respectively. *θ* is defined as the deflection angle of the load position to the fixed end of the cantilever. **d**, Sequential deflection images of the cantilever picospring fabricated at 5.5 mW under increasing loads. Inset: finite element analysis results. Scale bar, 10 μm. Source data

**Extended Data Fig. 3:**
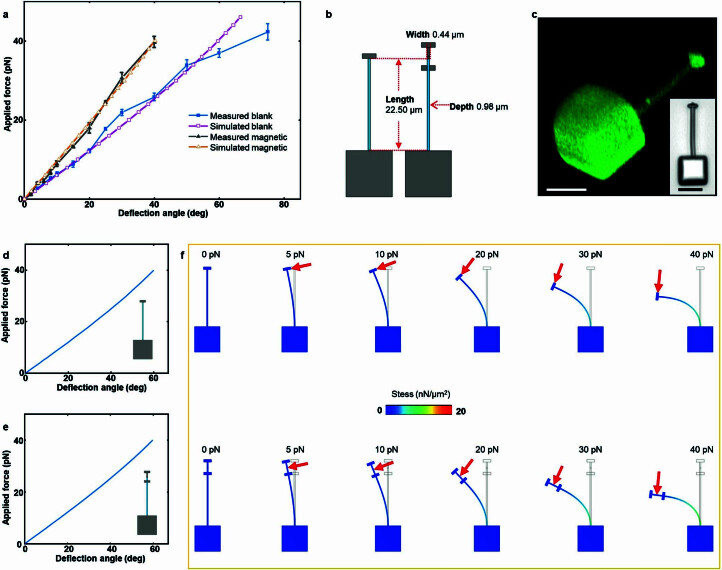
Comparable force responses of the cantilever picospring for modulus characterization and the microforcemeter. (**a**) Deflection curves of the cantilever picospring according to experimental results and FEA results. Comparison of blank samples between measured and simulated curves: F value = 0.0081 (ANOVA), Pearson correlation coefficient = 0.9926. Comparison of the magnetic samples between measured and simulated curves: F value = 0.0038 (ANOVA), Pearson correlation coefficient = 0.9984. Measured data are presented as mean ± s.d. (n = 3 picosprings). (**b**) Comparable geometric characteristics of the microforcemeter used for force measurement (left) and the cantilever picospring used for modulus characterization (right). (**c**) 3D-reconstructed image of the microforcemeter taken by confocal laser scanning microscopy (CLSM). Inset: optical image. (**d**) and (**e**) Highly consistent bending curves of the microforcemeter and cantilever picospring according to FEA simulations. (**f**) Simulated deflections of these two devices under loads. Source data

The local elasticity of the material depends on the cross-link density^28^ and thus is determined by exposure dose. We characterize the material elasticity based on a classic cantilever method by using optical traps (Fig. 2a,b). Under the same trapping power, cantilevers fabricated at lower laser powers present higher deflections. As shown in Fig. 2c, Young’s modulus of the elastomeric material already increases over 5-fold, increasing the laser power from 5.00 mW to 6.25 mW, and over 200 times up to 25 mW (Supplementary Fig. 3), which allows for a wide programming range of the elasticity for the picospring material by locally programming the laser power. This mechanical characterization result is universally applicable to all microstructures based on the present elastomeric material. Owing to the reproducibility of high-resolution TPL, picosprings fabricated at the same exposure parameters show no significant mechanical difference. The magnetic-elastomeric material is less compliant than the non-magnetic-elastomeric material due to the doping with the rigid MNPs. Figure 2d and Supplementary Video 1 show the real-time deflection of the cantilever picosprings under increasing forces and simulated results using the measured modulus. Experimental results agree well with simulated results for large deformations up to 75° (see also Extended Data Fig. 3a).

## Picosprings as real-time piconewton-scale force indicators

**Fig. 3:**
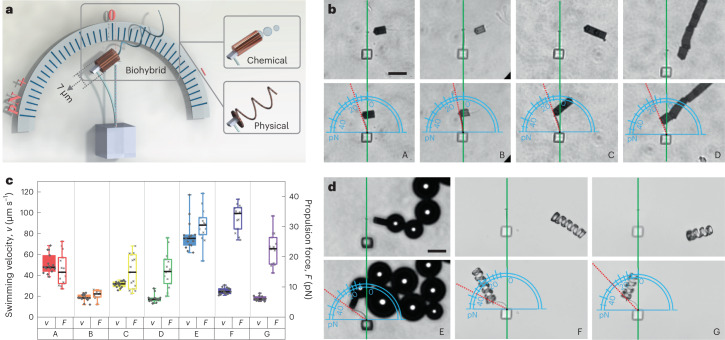
Picospring as a real-time indicator for piconewton-scale forces. **a**, Schematic illustration of the microforcemeter application in measuring the propulsion forces of different types of microswimmer: tubular sperm–motor (biohybrid), actuated by the sperm flagella; microjet (chemical), actuated by the generated bubbles; microhelices (physical), actuated magnetically. All microswimmers are guided magnetically. The microforcemeter inherits the mechanical properties and fabrication accuracy of the cantilever picospring employed for the characterization purpose above, resulting in a high measurement consistency. The force-sensing range and limit are both tunable by the picospring configuration and the laser power of TPL. We use the photoresist without MNPs to fabricate the microforcemeter, to avoid the influence of the magnetic field on the measurement accuracy. **b**, Microforcemeter deformation under the propulsion force of: A, one-tube sperm–motor; B, one-tube sperm–motor at 25 °C; C, two-tube sperm–motor; D, six-tube sperm–motor. All microswimmers except B were measured at 37 °C. **c**, Peak propulsion forces and swimming velocities of different microswimmers (*n* =15 microswimmers for each group). Boxes plot minimum, first quartile, median, third quartile and maximum values. The sperm–motor trains, consisting of multiple tubes, show no significant difference from the one-tube sperm–motor in the propulsion force measured at a speed of 0 μm/s. This is consistent with the fact that the elastic force of the cantilever picospring accurately equals the propulsion force provided by the sperm when there is no fluid drag (otherwise the drag force is typically proportional to velocity with a coefficient of friction determined by geometry). Thus, in the static state, the influence of the volume and friction of the synthetic part of the sperm–motor can be neglected on the picospring-based measurement of the sperm’s propulsion force. **d**, Microforcemeter deformation under: E, chemical microjet; F, long microhelix; G, short microhelix. A longer microhelix has higher propulsion force and swimming velocity under the same rotating magnetic field. This is consistent with the increased frictional drag for the longer helix. Scale bar, 20 μm. Source data

The energy stored in the picospring critically depends on its deformation under load. Hence, the applied force on the picospring can be quantified intuitively by its deflection. Here we present a microforcemeter based on a cantilever picospring to demonstrate its real-time function of indicating dynamic biological forces at the piconewton scale, testing it in connection with propulsion forces of microswimmers (Fig. 3a). The microforcemeter is calibrated by obtaining its force–deflection curve (Extended Data Fig. 3b–f). Superior to the previously reported hydrogel microfibres^29^ or polydimethylsiloxane micropillars^30^, used for characterizing static forces of adherent cells, our microforcemeter can be fabricated with better adapting 3D configurations to tackle the complicated movement of different microswimmers, ranging in type from biohybrid (for example, sperm–motor) to chemical (for example, microjet) to physical (for example, magnetic microhelix)^31^.

In situ monitoring of the real-time propulsion force is crucial for guiding the design of artificial microswimmers^32^ as well as for exploring the interaction between natural microswimmers and the human body, such as the membrane penetration mechanism of sperm during fertilization^19^. Figure 3b (A) and Supplementary Video 2 show the microforcemeter deformation under the action of a tubular sperm–motor. At low Reynolds number, the propulsion force is balanced by the fluid drag and the elastic reaction force from the cantilever. When the sperm–motor speed decreases to 0, the maximum deflection angle of the cantilever directly reads the propulsion force, 15.5 ± 5.0 pN at 37 °C or 7.0 ± 1.5 pN at 25 °C (Fig. 3b (A and B) and Fig. 3c), in agreement with the reported values obtained by other techniques^33,34^. During the contact period, the fluctuating deflection of the microforcemeter tracks the real-time beating of the sperm flagellum. The sperm–motor speed changes inversely with the elastic force of the cantilever (Supplementary Fig. 12), reflecting the conversion between the kinetic energy of the sperm–motor and the elastic potential energy of the picospring.

As shown in Fig. 3c (E), the microjet shows significant fluctuations on the measured propulsion force over time, which closely corresponds to the bubble generation from its tail. When the microjet makes contact with the microforcemeter, the microcantilever responds by bending as the bubble enlarges. Subsequently, when the bubble size decreases, the microcantilever ceases deforming or even begins recovering until the next bubble reaches the appropriate size. The chemical microjet and the sperm–motor with comparable geometric features show similar values on the ratio of the propulsion force *F* to the swimming velocity *v*, in line with the low Reynolds number Stokes equations for geometry-dependent hydrodynamic friction *F* *∝* *v* (ref. ^32^). In contrast, the magnetically driven microhelix under corkscrew-like motion (Fig. 3d (F and G)) has a much higher propulsion force than the sperm–motor with a similar swimming velocity, indicating less efficient energy conversion of the helical structure. The microhelices and other microswimmers create fluid flows around them during motion. The microforcemeter can effectively capture the deformation of the surrounding fluid, enabling real-time measurements of forces involved in liquid–solid interactions.

## Picospring for initiating gripping action

**Fig. 4:**
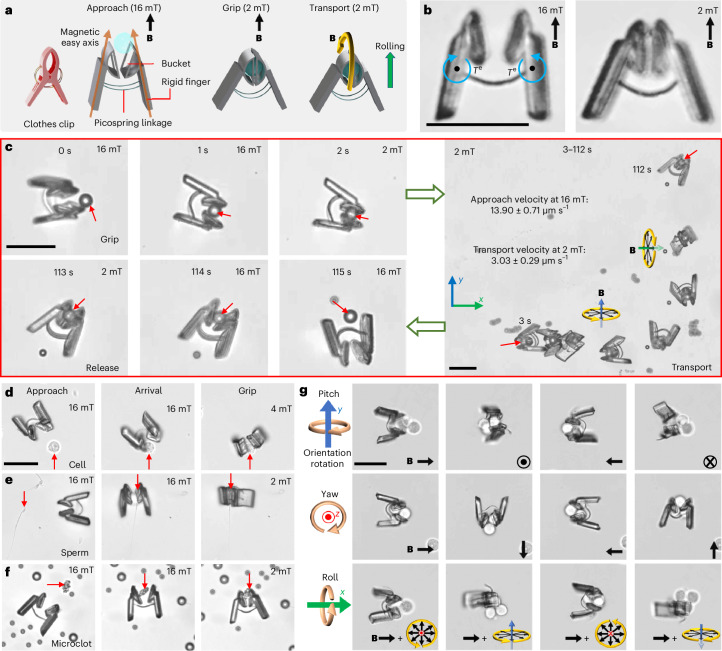
Super-compliant picospring for initiating gripping action on cellular objects. **a**, Schematic illustration of the microgripper’s working process. **b**, Opening and closing images of the microgripper. *T*^e^, elastic torques from the deformed arc picosprings initiating the closing of the microgripper bucket. **c**, Time-sequential images showing the grip, transport and release of a microbead. Red arrows point at the targeted microbead. Black arrows indicate the rotating magnetic field and yellow circular arrows indicate the rotation directions of the magnetic field vector. The microgripper performs a rolling motion to approach the object under a rotating magnetic field of 16 mT. After the microgripper arrives at the target (for example, a 5 μm microbead), the magnetic field is decreased to 2 mT to decrease the magnetic torques. The elastic torques of picosprings draw the gripper bucket shut with the microbead cargo inside (0–2 s). After picking up the microbead, the microgripper transports it in a rolling motion mode under a rotating magnetic field of 2 mT (3–112 s). In the end, the microgripper releases the object by simply opening its fingers again (113–115 s) as the 16 mT magnetic field is reapplied. **d**–**f**, Object-adapted gripping control strategies in three stages (approach, arrival and grip): gentle gripping of a sensitive cancer cell (**d**); firm enclosing of a sperm head (**e**); firm clamping of a waste-mimicking microclot (**f**). **g**, Orientation manipulation of a three-cell cluster along all three axes in space. From top to bottom: pitch, yaw and roll. The brown circle arrows indicate the rotation directions of the cell cluster orientations. Scale bar, 40 μm.

Benefiting from its force-indicator capability, the picospring can also provide gentle force, adjustable according to its degree of deformation, to initiate complex mechanical actions. Previously, microscale grippers typically relied on intense stimuli induced by physical or chemical environmental changes, which are challenging to achieve in deep tissue^35–37^. Magnetically controlled grippers were mainly developed at larger scales (0.3–1 mm)^38^. Here we demonstrate a clip-like magnetic microgripper with a length of 40 μm for single-cell manipulation by using picosprings as an elastic self-closing end effector as shown in Fig. 4a,b. Its picosprings take the form of arcs to gain higher angular deflection within a limited length. Under a high magnetic field, for example, 16 mT, the magnetic torques align the magnetic easy axes of the magnetic microgripper fingers towards the field direction, opening the microgripper bucket. It is gradually closed when the magnetic field decreases. Figure 4c and Supplementary Video 3 show the grip and transport of a 5 μm microbead. Previously, it was difficult for single-cell-scale robots to perform locomotion and gripping simultaneously using a single stimulus owing to the lack of a spring component. The present microgripper uses its rigid fingers as the magnetic actuation mechanism to open its bucket and facilitate the locomotion, while it uses its arc picosprings as the elastic actuation mechanism to initiate the gripping action. The multimodal control is realized by switching the magnetic fields between rotating and uniform.

One key problem in cell manipulation is how to hold the delicate target cell firmly enough without applying damaging stress to it^39^. In our previous studies on microrobots based on mechanical^40^ and fluidic^2^ traps, we encountered challenges in maintaining a balance between the stable transport and easy release of the cargo, particularly in complex fluidic environments. The compatibility of the present picosprings with 3D nanofabrication enables the design of a unique bucket structure controlled by the picospring latches, providing an efficient combination of stable transport and triggered release. Compared with simple clamping, enclosing sensitive objects (for example, live cells) in a bucket not only avoids high friction and pressure exertion but also protects the objects from external harm or getting lost on the way. In addition, the adjustable deformation of the microgripper facilitates stable but gentle gripping for objects of various sizes in different geometries. For example, with its maximum opening angle under the magnetic field of 16 mT, the microgripper can accommodate a 20 μm spherical target, exerting a maximum clamping force of 34.5 pN (Supplementary Fig. 14c,d).

When dealing with a HeLa cell at about 10 μm, as shown in Fig. 4d and Supplementary Video 4, the microgripper can gently close its bucket at a moderate angle under a moderate magnetic field (4 mT), applying a very low force of about 2.5 pN on the delicate cell (Supplementary Text 3). When dealing with a sperm cell with an approximately 3-μm-wide head and 40-μm-long tail (Fig. 4e), the microgripper can enclose the sperm head in its tightly closed gripper bucket and leave the tail outside ensuring stable transport. The ability to handle irregularly shaped objects may be useful in many practical applications. There are many such objects to be cleaned away in medical applications, such as travelling emboli^41^, microplastics retained in the body^42^ or other exotic wastes, in which cases, the microgripper can act as an in situ cleaner. As shown in Fig. 4f, the microgripper can be controlled to tightly clamp and stably transport an irregular microclot (see fabrication in Methods) among regular microbeads that mimic the cells in the oviduct fluid. Owing to the decoupled control of the locomotion and gripping actions, the microgripper can realize the multi-degree-of-freedom control of not only a single cell in different shapes but also a cluster of multiple cells. As demonstrated in Fig. 4g, the microgripper grips a three-cell cluster and precisely adjusts the orientation thereof along all three axes in space.

**Extended Data Fig. 4:**
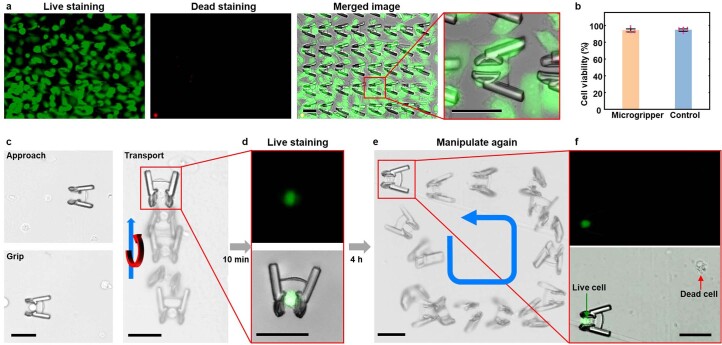
Biocompatibility test of the microgripper. (**a**) Live/dead staining of HeLa cells after 48 hours of co-culturing with the microgripper array. Green: Live cells stained by FDA with the emission wavelength of 530 nm; Red: Dead cells stained by PI with the emission wavelength of 618 nm. (**b**) Comparable cell viability of the HeLa cells after 72 hours of culturing on the microgrippers and commercial cell-culture substrate. Dot plots represent 6 individual samples for each group. Bars represent mean ± s.d. (**c**) and (**d**) Manipulation of a live HeLa cell by the microgripper and the subsequent live staining demonstrating the cell′s viability after the manipulation process. (**e**) Manipulation of the gripped HeLa cell in a rectangular trajectory after 4 hours of co-culturing. (**f**) Fluorescent images of the observed HeLa cell after 4 hours indicating the harmless interaction of the microgripper with the cell. Blue arrow and blue curved arrow in the motion-sequential images indicate the transport direction of the microgripper. Red curved arrow indicates the rotating direction of the microgripper. All scale bars: 40 μm. Source data

The magnetic field stands out as one of the most promising physical actuation mechanisms for in vivo applications of microrobots^31^. Its high tissue penetrability has been proven to be safe below 8 T (ref. ^43^), with typical driving magnetic fields for microrobots remaining below 100 mT. However, the use of laser-driven microrobots presents challenges in balancing tissue penetrability and safety. Although focused laser beams are promising in general for single-point and multi-point control, ensuring their safety remains a considerable concern due to the potential damage such as that arising from the photothermal effect^21^. It is important to note that neither the locomotion nor gripping of the present microgripper needs additional stimulation by drastic changes of the environmental conditions. The microgrippers show no discernible impact on the growth or viability of cells after co-culturing for 72 hours as illustrated in Extended Data Fig. 4a,b. Owing to the controllable gripping force and the high biocompatibility, the target cell is not affected by either the manipulation or the co-culturing for four hours when being gripped (Extended Data Fig. 4c–f and Supplementary Video 5). This manoeuvrable and safe strategy for manipulating cellular objects represents a promising route to in situ cell transplantation and gamete/zygote intrafallopian transport^44^.

## Picospring for actuating microrobots

**Fig. 5:**
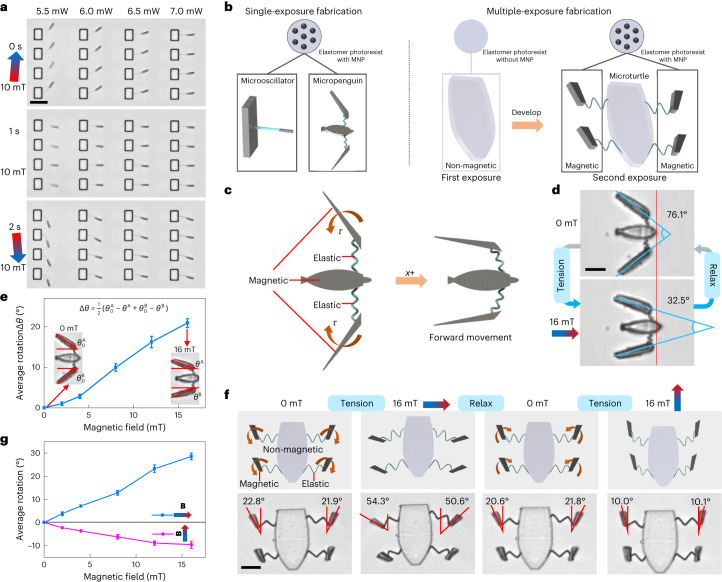
Super-compliant picospring to actuate microrobots. **a**, Microoscillator oscillation driven by the magnetic field. The deflection decreases from left to right as the cantilever picospring fabricated at higher laser powers becomes less compliant. The microoscillators oscillate over 100 times with no observable fatigue, revealing the structural stability of the picosprings. Scale bar, 30 μm. **b**, Single- or multiple-exposure method for fabricating micromachines with homogeneous or inhomogeneous magnetization distributions. **c**, Schematic illustration of the micropenguin propulsion generated by the magnetic torques (*τ*) that close the flippers. **d**, Closing moment of the micropenguin flippers. The picospring linkages store energy during the closing of the flippers. Under a magnetic actuation field (for example, 16 mT) along its axial direction (+*x*), the micropenguin closes its flippers and deforms the picospring linkages, achieving a forward movement by pushing the fluid backwards. The driving force is provided by the magnetic torque aligning the magnetic easy axis of each geometrically anisotropic flipper towards the magnetic field direction. When the magnetic field decreases to zero, the picosprings relax and drive the micropenguin back to its original position. Scale bar, 10 μm. **e**, Rotation angle (Δ*θ*) of the micropenguin flipper relevant to the magnetic field. $\theta_{0}^{\rm {A}}$, $\theta_{0}^{\rm {B}}$, *θ*^A^ and *θ*^B^ represent the angles between the axis of the micropenguin and its left or right wing at theinitial state ($\theta_{0}^{\rm {A}}$ or $\theta_{0}^{\rm {B}}$) and under specific magnetic fields (*θ*^A^ or *θ*^B^), respectively. The flipper rotation and the related picospring bending are positively related to the applied field. An approximately constant bending stiffness can be achieved in accordance with the bending model of a coil spring. **f**, Opposite magnetic torques acting on the microturtle flippers under orthogonal magnetic fields. The soft magnetic flippers can be aligned towards the orientation of the magnetic field but without direction selectivity. Each flipper has two rotation directions under different magnetic fields, whereas the non-magnetic torso’s orientation is not changed by the magnetic fields. Scale bar, 20 μm. **g**, Rotation angles of the microturtle flipper towards two magnetic field directions. For **e** and **g**, *n* = 3 microrobots for each group, mean ± s.d. Source data

**Extended Data Fig. 5:**
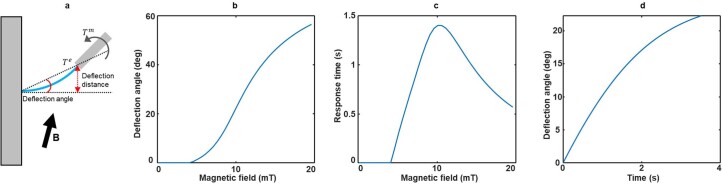
Modelling results of the deformation response of a microoscillator to the magnetic field. (**a**) Dynamic model of the microoscillator under a uniform magnetic field with a fixed direction. (**b**) Maximum deflection of the microoscillator with the increasing magnetic field. (**c**) Time for the microoscillator to reach 60% of its maximum deflection (response time). (**d**) Microoscillator deflection over time under a magnetic field of 10 mT.

In common with traditional springs, such as a bow or hairspring, the present picosprings can also power machines by the programmed release of stored energy. We start from the actuation of an array of microoscillators (Fig. 5a and Supplementary Video 6). Under an oscillating magnetic field, microoscillators with more compliant picosprings perform forced oscillation with higher amplitudes. Such simple devices are useful as fundamental building blocks for a variety of complex machines. As shown in the first row of the microoscillator array in Fig. 5a, a 15-μm-long spring can achieve a deflection of about 17.2 μm (approximately 38.3°) when driven by a 10-μm-long magnetic bar under a magnetic field of 10 mT. The response time of such microrobots depends on the stiffness of the picospring and the magnetic response of the driving component. As simulated in Extended Data Fig. 5, a typical microoscillator system takes about 1.4 s to reach 60% of its maximum deformation under a 10 mT magnetic field.

In addition, the magnetically driven oscillation indicates a direct way to measure the picospring stiffness based on the balance of the elastic and magnetic torques. This torque-based measurement is particularly useful for untethered micromachines (microrobots) that cannot be characterized by optical tweezers or other force-based measurement means. As shown in Fig. 5b, our magnetic-elastomeric material enables one to construct such complex picospring-based microrobots with onboard actuation mechanisms, for example, a fully magnetic ‘micropenguin’ (20 μm in length) with coil springs and a partially magnetic ‘microturtle’ (50 μm in length) with zigzag picosprings. These microrobots are designed to use their movable magnetic parts as the spring winder driven by the external magnetic field. Figure 5c,d and Supplementary Video 7 show the morphing of the untethered micropenguin. The micropenguin achieves a forward movement after closing its flippers by the magnetic torques induced by the magnetic actuation field (for example, 16 mT) and moves back after opening its flippers by the elastic torques from the deformed picosprings at 0 mT. Bending stiffness of the coil picospring can be deduced approximately from the correlation between the picospring deflection and the magnetic torque of the flipper, which are dependent on the flipper rotation and the magnetic field, respectively (Fig. 5e and Supplementary Text 4). Inhomogeneous magnetization distributions provide more complex morphing modes for the microturtle (Fig. 5f,g and Supplementary Video 8). The zigzag picospring linkages have two tensioned states occurring when the microturtle flippers enlarge or narrow their angles towards the lateral or axial field direction.

**Fig. 6:**
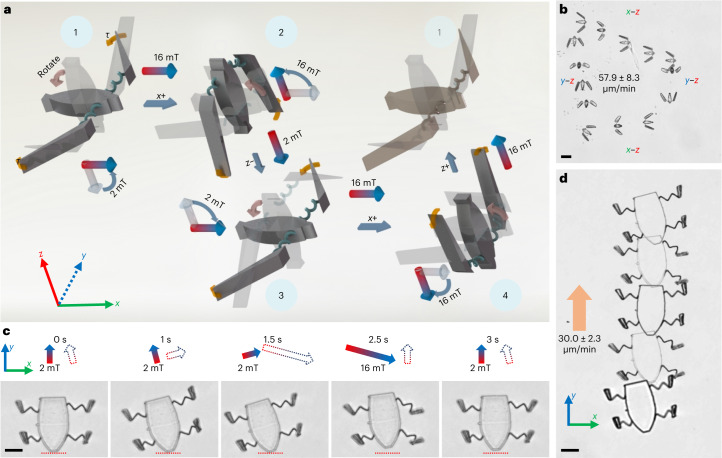
Locomotion of the picospring-based microrobots. **a**, Schematic snapshots of the micropenguin gait under an orientation-switching control strategy. The dark and transparent structures show the current and previous orientations of the micropenguin at a certain position, respectively. Yellow curved arrows indicate the magnetic torques (*τ*) that facilitate the flippers closing and opening. Red curved arrows indicate the micropenguin rotation. The magnetic torques under the magnetic actuation field (*B*_*x*_) close the micropenguin flippers to generate a forward movement (phases 1–2 and 3–4) while the elastic torques under the magnetic base field (*B*_*z*_) open the flippers for shape recovery (phases 2–3 and 4–1) after the micropenguin rotates 90° along the *y* axis. From phases 1–4, the micropenguin moves along the *+x*, *−z*, *+x* and *+z* directions sequentially. The base field (2 mT) is necessary to keep the micropenguin’s orientation. **b**, Stacked images of the micropenguin locomotion under the sequential magnetic field with a cycle duration of 5.5 s. **c**, Time-sequential images of the microturtle gait during one movement cycle. Red dashed lines indicate the starting position. Solid and hollow arrows show the respective magnetic field directions lasting till and starting from the current time points. The elastic potential energy stored in the *z* picosprings independently actuates the non-magnetic torso. The presence of the non-magnetic torso helps the microturtle to avoid undesired rotation of the whole body under the magnetic field. When the left/right picosprings actuate the microturtle forwards, the right/left picosprings buffer the rotation of the torso. When the magnetic fields cycle among 0°, 15°, −75° and −105° to the axial direction of the microturtle, the microturtle gains a net displacement by utilizing the phase difference between the rotations of its left and right flippers, owing to the programmed release of the stored energy in the picospring linkages. **d**, Locomotion sequence of the microturtle under a magnetic actuation field of 16 mT. The orange arrow indicates the locomotion direction. Scale bar, 20 μm.

**Extended Data Fig. 6:**
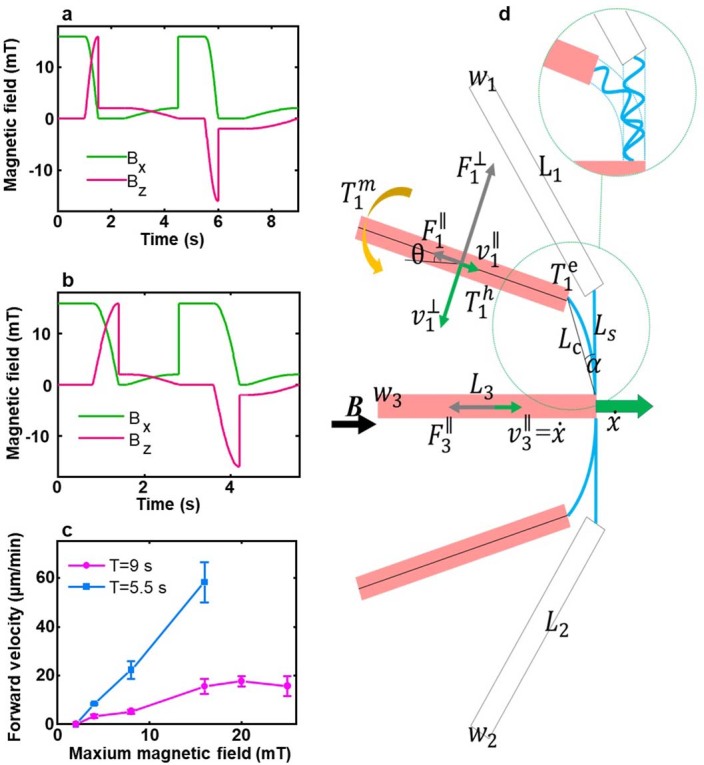
Orientation-switching control strategy for the micropenguin. (**a**) Magnetic field sequence in one cycle of 9 s at an actuation field of 16 mT. (**b**) More efficient control with simultaneous micropenguin rotation and flipper open/close processes with a cycle duration of 5.5 s. (**c**) Measured locomotion velocity of the micropenguin relevant to the magnetic actuation field with two cycling durations of 9 and 5.5 s (n = 3 micropenguins for each group, mean ± s.d.). (**d**) Dynamic model of the micropenguin. ${w}_{i}$: segment width; ${L}_{i}$: segment length; ${L}_{c}$: Chord length of the spring; ${L}_{s}$: Length of the spring connecting rod; ${T}_{i}^{h}$ (resp. ${T}_{i}^{m}$ *and* ${T}_{i}^{e}$): Magnetic torque (*resp*. elastic torque and hydrodynamic torque) on segment *i*; ${F}_{i}^{\parallel }$ (${resp}.\,{F}_{i}^{\perp }$): Hydrodynamic drag parallel (*resp*. perpendicular) to the long axis of segment *i*: ${v}_{i}^{\parallel }$ (${resp}.\,{v}_{i}^{\perp }$): the projection of the velocity of segment *i* on the direction parallel (${resp}.$ perpendicular) to the axis of the segment. *θ*: Orientation angle of the fin; *α*: Deflection angle of the elastic linkage; $\dot{x}$: Locomotion velocity of the torso. Source data

**Extended Data Fig. 7:**
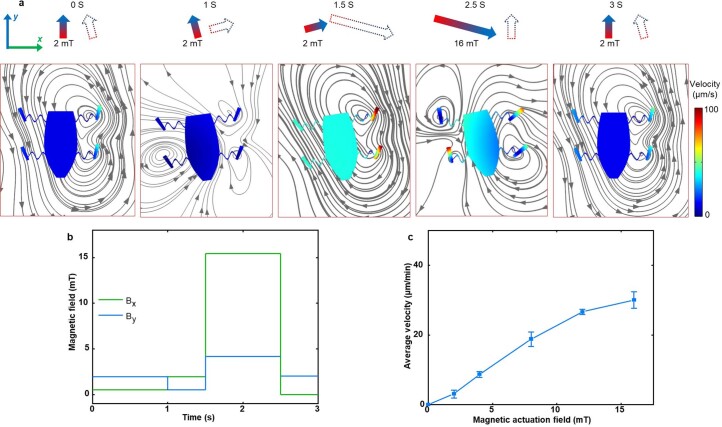
Sequential-motion control strategy for the microturtle. (**a**) Time-sequential images of the microturtle locomotion under a sequential-motion control strategy based on FEA results. Arrow lines illustrate the streamlines of the fluid. Solid and hollow arrows show the magnetic field direction lasting until and starting from the current time points, respectively. (**b**) Magnetic field sequence in one control cycle with a cycle duration of 3 s. When the magnetic fields cycle among 0°, −15° and 75° to the axial direction of the microturtle, the microturtle gains a net displacement, thanks to the programmed release of the stored energy in the picospring linkages. While the microturtle flippers are responsive to the magnetic field, its large non-magnetic torso is only controlled by the elastic torques from the deformed picosprings, stabilizing the microrobot movement. With the help of the buffering of the picosprings, the left and right pairs of flippers can perform asymmetric movement when the magnetic field direction changes. Hence, by alternating the magnetic actuation field (16 mT) and base field (2 mT) in different directions, the microturtle can gain a net displacement along its axial direction (*y-*axis) but with a reciprocal motion along its lateral direction (*x-*axis). (**c**) Locomotion velocity of the microturtle containing 5% MNPs with respect to the magnetic actuation field (n = 3 microturtles, mean ± s.d.). Source data

The picospring system serves as an onboard actuator for soft microrobots by converting the magnetic potential energy steadily to kinetic energy countering fluidic dissipation. Figure 6a illustrates the orientation-switching strategy to control the micropenguin. The micropenguin generates a net displacement by periodically switching its orientations when flippers close and open under 16 mT and 2 mT, reaching a velocity of 15.5 ± 3.1 μm/min or 57.9 ± 8.3 μm/min with a cycle duration of 9 s or 5.5 s (Fig. 6b and Extended Data Fig. 6). The picospring, as a soft actuator, can also enable microrobot locomotion by only shape morphing in two dimensions, without out-of-plane movement. As a demonstration, we present a sequential-motion strategy to control the partially magnetic microturtle (Fig. 6c,d). Here a set of picosprings act alternately as actuators and buffers. The left and right pairs of flippers can thus perform asymmetric movement when the magnetic field direction changes. By alternating the magnetic actuation field (16 mT) and base field (2 mT) in different directions (for example, as depicted in Extended Data Fig. 7), the microturtle can be actuated sequentially to gain a net axial displacement (*y* axis) but with a reciprocal motion along its lateral direction (*x* axis), reaching a maximum velocity of 30.0 ± 2.3 μm/min at 16 mT. Working parameters are derived from dynamical models (Supplementary Texts 4 and 5).

**Extended Data Fig. 8:**
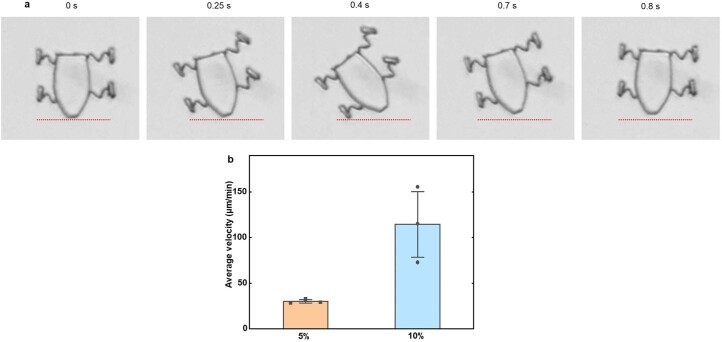
Microturtle velocities. (**a**) Locomotion gait of the microturtle containing 10% MNPs. The microturtle containing 10% MNPs exhibits a movement cycle of 0.8 s without stepping out, whereas the microturtle containing 5% MNPs takes 3 s for one movement cycle. Red dashed lines indicate the starting position. (**b**) Locomotion velocities of two types of microturtles under the magnetic field of 16 mT. Dot plots represent 3 individual samples for each group. Bars represent mean ± s.d. Source data

The locomotion efficiency of small-scale soft robots is indeed restricted by the rates of deformation and elastic recovery^45^. In our study, the microturtle’s movement can be decomposed into four processes as shown in Fig. 6c. Although the microturtle gains a peak propulsion velocity of about 6 μm/s within 1.5–2.5 s, it experiences some backwards movement during the recovery process (2.5–3 s). As a result, the average forward velocity (30.0 ± 2.3 μm/min) is relatively slow after consuming much time on deformation recovery. Optimizing the structural design or increasing the actuation magnetic field are promising strategies to improve the locomotion velocity. However, the most effective approach is to increase the magnetization of the microrobot itself. As shown in Extended Data Fig. 8 and the 3rd chapter of Supplementary Video 8, the microturtle with double concentration of MNPs achieves an actuation frequency of 1.25 Hz without stepping out, resulting in an increased locomotion velocity of 114.5 ± 41.3 μm/min. However, the increase in MNP concentration affects the uniformity of the 3D fabrication, leading to reduced motion stability.

## Conclusions

We have presented the 3D nanofabrication of picospring-based soft microrobots with programmable elasticity distributions. The picosprings perform micrometre-scale deformations that can be directly used to control complex actions of micromachines at the single-cell scale in biological conditions. The picospring system can recover a large bending of over 99% to its length (85°) over 100 times, ensuring high operation stability. Picosprings in different configurations tackle diverse tasks. The microforcemeter represents a simpler and more intuitive technique for the in operando measurement of ultralow forces of single cells compared with conventional bulky instruments, such as the atomic force microscope (AFM)^33^ and optical trap^34,46^. This picospring-based method can directly reveal the in situ response of a microswimmer interacting with a potential physical obstacle, such as an oocyte membrane or a blood clot. In such stationary measurements, the viscoelastic drag on the microforcemeter is negligible. The microforcemeter bending accurately reflects the microswimmer propulsion force in the specific media. As an example, the microforcemeter can effectively indicate the enhanced propulsion of the rotating microhelix in more viscous liquids, as shown in Supplementary Fig. 12c.

Utilizing the correlation between the picospring deformation and its output elastic force, we have developed a purely mechanical microgripper with adjustable gripping power for manipulating single cells. The deformed picospring can provide adjustable stresses between 0 and 34.7 mPa (Supplementary Text 3). The microgripper can perform not only the reversible gripping but also the six-degree-of-freedom locomotion controlled only by the magnetic field, which is of practical importance for biomedical operations, owing to the high tissue penetrability and biological safety of the magnetic field^47^. The gripping action is initiated by the picospring with no need for other stimuli such as pH or temperature change, which are relatively harmful to susceptible cells^48^.

Beyond the on–off control of gripping action, the present picospring system is capable of continuously powering untethered microrobots. Previously, small-scale soft robots with multimodal locomotion have proven advantageous in their adaptability to unstructured environments^12,49,50^. Here a set of multiple picosprings enable complex motion modes for soft microrobots with lengths down to 20 μm, aiming at the applications in the smallest body cavities. For future applications, a magnetic microrobot integrated with the sensor, actuator and operating components could be employed in drug delivery and microsurgery in vivo. Picosprings, with their ultralow-force deformation, are promising for constructing new flexible machines, such as on-chip AFM, microaccelerators and microbiopsy forceps.

## Methods

### Magnetic-elastic photoresist preparation

All chemicals were purchased from Sigma-Aldrich unless otherwise specified. The elastic photoresist consisted of urethane acrylate oligomer 70 wt%, poly(ethylene glycol) diacrylate 28.40 wt% as the crosslinker, 1-(4-(2-(dimethylamino)ethoxy)phenyl)-2-phenyl-1-butanone 1.5 wt% as the photoinitiator, and a complex of 2,2,6,6-tetramethylpiperidine-1-oxyl 0.05 wt% and methyl methacrylate 0.05 wt% as the quencher. The mixture was bubbled with nitrogen for 30 min and vacuumed for 30 min to degas. MNPs were prepared based on a classic coprecipitation method. Briefly, 5.38 g FeCl_3_·6H_2_O and 1.98 g FeCl_2_·4H_2_O were dissolved in 200 ml H_2_O. Then 7 ml 25% ammonium hydroxide was dropped in the mixture, which was continuously stirred for 3 h. The collected particles were then washed with water three times and further modified by 3-(trimethoxysilyl)propyl methacrylate in ethanol at concentrations of 1 wt% and 0.5 wt% at 80 °C for 1 h (ref. ^20^). MNPs were collected after washing with ethanol three times. The magnetic-elastic photoresist was prepared by mixing MNPs into the elastic photoresist at a concentration of 5% or 10% for the special microturtle containing double concentration of MNPs. Finally, the magnetic-elastic photoresist was bubbled with N_2_ for 30 min and vacuumed for 30 min. Prepared photoresist should be always kept from light at 4 °C before use.

### Numerical analysis

To design the microstructures efficiently based on the material properties, simulations were performed to predict the shape morphing of the microstructures before fabrication. For the results presented in Figs. 1d and 6d, and Extended Data Figs. 3 and 7, we used a user-defined multiphysics module of the commercial finite element analysis software Comsol. All solids and fluids were regarded as incompressible. Young’s modulus *E* was set as 0.422 MPa for the microforcemeters and 1.525 Mpa for the other elastic components, according to mechanical characterization results of the cantilever picospring. The Poisson ratio for all materials was set as 0.49, assuming that the material is quasi-incompressible. In all simulations, the sperm medium (SP-TALP) was set as a Newtonian fluid with the density of 10^3^ kg m^−3^ and viscosity of 1 mPa s. During finite element analysis, the applied load was given as a function of the magnetic torque in the local coordinate system. The magnetic torque *T*^m^ was calculated by using a simplified function applied to the soft magnetic material^51^:

$$\begin{array}{l}{T}^{{\mathrm{m}}}=\frac{\chi V}{\mu }{B}^{2}\,\sin \left(\theta -\arctan\left(\tan\theta \times \frac{1+0.118\chi }{1+0.432\chi }\right)\right)\\\sqrt{{\left(\frac{\cos\theta }{1+0.118\chi }\right)}^{2}+{\left(\frac{\sin\theta }{1+0.432\chi }\right)}^{2}}\end{array}$$

where *θ* is the angle from the magnetic field with a flux density of *B* to the easy magnetic axis of the segment; *χ*, *V* and *μ* represent the magnetic susceptibility and bulk volume of the segment, and the magnetic permeability of water (see details in Supplementary Text 2). The boundary loads of the mechanics simulation were applied parallel to the cross-section of the elastic springs in the local coordinate system. The magnetic torques applied on the flippers of the microturtle shown in Fig. 6d were calculated according to the equation above, by simplifying the flippers as rectangular shapes as projection in two dimensions.

The micropenguin was furthermore analysed with a kinematic model solved by the Runge–Kutta fourth-order iterative method with MATLAB. As shown in Extended Data Fig. 6d, the micropenguin flippers and torso were simplified as cuboids. The elastic components were simplified as linear springs. The bending stiffness of the elastic component was obtained by fitting the balanced magnetic torque with respect to the deflection angle, which is measured as half of the varied angle of two flippers at each magnetic field. Additional simulation parameters can be found in Supplementary Text 2. The simulation results were then used to guide the design and fabrication of the microstructures, and were furthermore validated by the experimental results.

### Microstructure fabrication

Microstructures were fabricated by using a 3D direct laser writing system (Photonic Professional GT, Nanoscribe). During the fabrication, the laser power was set as 25.0 mW for all rigid parts, 5.5 mW for the force-sensing picosprings and 6.0 mW for all the other elastic components, unless otherwise specified. After exposure, the sample was developed in acetone for 24 h to remove all unpolymerized components. As shown in Extended Data Fig. 1a, the environment was changed from acetone to water-based media with pluronic acid F127 (PF127) as a thickener gradually at a rate of 200 μl min^−1^ for 12 h. After that, the solution was gently replaced with SP-TALP by pipette. Structural integrality of the picospring-based microstructures was well kept after these operations (Extended Data Fig. 1b). Notably, in the microgripper experiment, SP-TALP was furthermore replaced by a cell media mimicking oviduct fluid (cell media containing 0.4% methylcellulose)^52^.

During the fabrication of the microoscillator, the coil-spring microoscillator and the microforcemeter, the glass substrate was silanized before use to avoid the detachment of the microstructures from the substrate. 3-(Trimethoxysilyl)propyl methacrylate was used to attach methacrylate terminal groups onto the substrate, forming a covalent linkage between the glass substrate and the magnetic-elastic photoresist^53^.

During the fabrication of the microturtle, the exposure was performed twice by using the photoresist with and without MNPs. First, the elastic photoresist without MNPs was used to fabricate the torso. After that, the photoresist was replaced with magnetic-elastic photoresist. The glass substrate was glued with a glass capillary as an aligning indicator to be aligned to the previously marked tick lines on the sample holder to align the sample to the same position as the first exposure. The origin was found again based on the position of the fabricated torso and the structure code was corrected with a specific angle based on the orientation change of the torso to maximally enhance the fabrication accuracy. Then the second exposure was performed to fabricate the flippers and elastic components.

### Material characterization

A confocal laser spectrum microscope (Zeiss LSM 980) was used to obtain the 3D geometry of the microforcemeter at excitation laser of 488 nm and emission detection of 580 nm. ImageJ was used to generate the 3D model of the structure and measure the dimensions.

The elastic property of the cantilever was calibrated by an optical trap system (Lumicks C-Trap). Five-micrometre polystyrene microbeads were used to calibrate the laser power of the optical trap, giving the trapping force constants of certain laser powers. Microbeads were then pulled to deform the microforcemeter as slowly as possible, so that the drag force could be neglected. The bending curve of the microforcemeter with respect to the applied force can then be determined by recording the positions of the microbead and the deflection angles of the cantilever (see details in Supplementary Text 1.2). Each group of measurements was repeated on three samples. Images and videos were analyzed with ImageJ and data were fitted with OriginPro. The viscosity of SP-TALP was taken as 1 mPa s. The mechanical characterization of the rigid parts fabricated at 25 mW was done using an AFM, shown in Supplementary Fig. 3 (see details in Supplementary Text 1.2).

The magnetization property of the material was characterized by a superconducting quantum interference device magnetometer (SQUID, Quantum Design) at room temperature with magnetic fields up to 100 mT. The samples were prepared as an array of 8,848 rectangular solids with a length of 15 μm and sectional area of 16 μm^2^. The volume susceptibility was calculated as 0.1220, by fitting the magnetization with respect to the applied field using OriginPro software.

### Propulsion force measurement by the microforcemeter

The sperm–motor microtubes, tubular microjets and microhelices were all fabricated by TPL using IP-DIP as photoresist. After exposure, the samples were dried in a critical point dryer after 20 min of development in mr-Dev 600 (Micro Resist) and washed three times with isopropanol. Metal layers of Fe (10 nm)/Ti (5 nm) were coated on the sperm–motor microtubes and the microhelices by sputtering. Layers of Fe (10 nm)/Ti (5 nm)/Pt (10 nm) were coated on the tubular microjet by e-beam deposition. Bovine sperm were prepared following the previously reported protocol^2^. All samples were treated in PF127 solution (1%) for 0.5 h before use. The measurement of the sperm–motors was performed in the microforcemeter chamber with 1 ml SP-TALP containing about 10^3^ microtubes and 10^4^ sperm. The sperm–motor was formed when a sperm became constrained in a microtube by randomly swimming. The sperm–motor was then guided by the external magnetic field, at around 2 mT, towards the action bar of the microforcemeter. The magnetic field was adjusted perpendicularly to the action bar, to avoid the influence of the magnetic torque on the cantilever deformation. The measurement of the microjets was performed in SP-TALP containing 1% H_2_O_2_ and 0.1% sodium dodecyl sulfate. Approximately 20 microjets were added and guided in the same way as the sperm–motors. The measurement of the microhelices was performed by applying a rotating magnetic field of 10 mT at 40 Hz for magnetic actuation. The propulsion force, that is, the elastic force when the sperm–motor speed is zero, was calculated by linear interpolation in the calibration curve of the microforcemeter except for the propulsion force of the microjet, which was obtained from the finite element analysis simulation curve of the short microforcemeter. All measurements were done at 37 °C unless otherwise specified. Videos and data were analysed by ImageJ and OriginPro. Elastic forces were calculated by interpolation in the microforcemeter calibration curve in Fig. 3c,d.

### Magnetic control of the microgripper

The magnetic actuation was performed by an electromagnet system (Magnebotix MFG 100-i). The time-sequential magnetic fields were generated by designing *B*_*x*_, *B*_*y*_ and *B*_*z*_ with piecewise functions. After the media changing process, the microrobot and microgripper samples were treated in the ultrasonic bath for 5 min. Then a 100 μl pipette was used to gently blow the samples with media to fully detach the microstructures from the substrate without silanization. In the experiments of microrobots, the samples were then directly dispersed in SP-TALP and operated in the magnetic field. In the experiments of the microgripper, the sample solution was added with pre-prepared microobjects (microbeads and microclots). The microbead sample was obtained by directly dispersing 5 μm polystyrene microbeads at about 10^3^ ml^−1^ as shown in Fig. 4c,f. The protein-based microclots were synthesized with bovine serum albumin by using a microemulsion method as reported previously^2^. The oviduct-fluid-mimicking solution was prepared based on the HeLa cell media containing 0.4% methylcellulose to mimic the viscoelastic property of the fluid. Rotating magnetic fields were applied for the locomotion of the microgripper in a rolling manner and uniform magnetic fields were applied to open the gripper bucket. Videos and data were handled with ImageJ and OriginPro.

After manipulating the HeLa cells, the target cell was stained by a live/dead staining kit containing fluorescein diacetate and propidium iodide. Following a 10 min incubation period, multi-channel fluorescence images were captured using excitation at a wavelength of 470 nm for live cells (emission wavelength 530 nm) and 540 nm for dead cells (emission wavelength 618 nm). Subsequently, the target HeLa cell was cultured inside the microgripper’s bucket for an additional 4 h. A second manipulation was then performed to transport the HeLa cell along a rectangular trajectory. After this manipulation, fluorescence images were once again acquired. The presence of green fluorescence of the target cell after manipulation indicated that the microgripper had no adverse impact on the cell’s viability during manipulation, in contrast to the red fluorescence observed in randomly dead cells. The control of cell orientation shown in Fig. 4g was implemented by changing the direction of the applied magnetic field vector after the microgripper had gripped the cell cluster. A uniform magnetic field of 6 mT was applied along *+x* direction to grip and define the initial orientation of the cell cluster. For changing the cell orientation in the *x*–*y* (yaw) or *x*–*z* (pitch) planes, the magnetic field vectors were simply rotated along the *z* or *y* axes by any degree on demand. For changing the cell orientation in the *y*–*z* plane (roll), another rotating magnetic field at 2 mT and 20 Hz was applied. The orientation of the cell cluster in the *y*–*z* plane was changed by changing the rotation axis of the rotating magnetic field, while the uniform magnetic field of 6 mT was kept along the *+x* axis.

### Magnetic control of the micropenguin and microturtle

Microrobots with time-symmetric motion cannot achieve a net displacement at low Reynolds number^54^. An efficient strategy to break time symmetry is to make the microrobot’s orientation during morphing different from its orientation during recovery. As a demonstration, we implement an orientation-switching strategy to control the micropenguin. Extended Data Fig. 6a depicts the sequences of the magnetic fields with a cycle duration of 9 s as shown in Fig. 6a: 0–1 s, a uniform magnetic field of 16 mT was applied along the *x* axis (phases 1–2); 1–1.5 s, rotation magnetic field of 16 mT along the *y* axis; 1.5–2.5 s, uniform magnetic field of 2 mT along the *z* axis (phases 2–3); 2.5–4.5 s, rotation magnetic field of 2 mT along the *y* axis; 4.5–5.5 s, uniform magnetic field of 16 mT along the *x* axis (phases 3–4); 5.5–6 s, rotation magnetic field of 16 mT along the *y* axis; 6–7 s, uniform magnetic field of 2 mT along the *z* axis (phases 4–1); 7–9 s, rotation magnetic field of 2 mT along the *y* axis. After a cycle of 9 s, the micropenguin recovers its original orientation and gains a net displacement along the *x* axis. Extended Data Fig. 6b shows the magnetic field sequences with a cycle duration of 5.5 s of the micropenguin in a more efficient swimming manner. In this case, the uniform and rotation magnetic fields were mixed, enabling simultaneous micropenguin rotation and flipper opening and closing.

One disadvantage of the orientation-switching control strategy is the concomitant rotation of the whole robot, despite its universal applicability to elastic microrobots for generating a net displacement. This rotation can be avoided by using a set of picosprings driving different movable parts of microrobots with inhomogeneous magnetization, for example, the microturtle. Extended Data Fig. 7a shows the finite element analysis simulation results, which help seek out the most efficient directions of the magnetic fields. The magnetic field sequence of the final control strategy is shown in Extended Data Fig. 7b. Only uniform magnetic fields are needed to generate a net displacement for the microturtle owing to the coordinated actuation and buffering functions of the left and right pairs picosprings controlling different flippers. The microturtle was then only controlled to move in two dimensions of the *x*–*y* plane with no rotation or displacement in the *z* axis: 0–1 s, 2 mT along 15° (anticlockwise direction as positive) direction from the *+y* direction (symmetric axis of the microturtle); 1–1.5 s, 2 mT along −75° from *+y*; 1.5–2.5 s, 16 mT along −105° along *+y*; 2.5–3 s, 2 mT along *+y*. All locomotion experiments of the microrobots were performed in PBS at 25 °C. The microturtle contains double concentration of MNPs was controlled with a cycling period of 0.8 s (Extended Data Fig. 8) with comparable phase sections of 0–0.25 s, 0.25–0.4 s, 0.4–0.7 s and 0.7–0.8 s.

### Biocompatibility evaluation

HeLa cells were used to assess the biocompatibility of the micromachines, specifically the microgripper arrays. In brief, 7 samples of fabricated microgripper arrays were placed in the cell culture wells of 6-well plates and filled with 3 ml of culture media. The control group wells were filled with only cell media. Each well was seeded with approximately 10^5^ HeLa cells. Following 48 h incubation, 1 well from the microgripper group and 1 from the control group were stained directly using the live/dead staining kit containing fluorescein diacetate (5 mg ml^−1^ in acetone) and propidium iodide (1 mg ml^−1^ in PBS). Multi-channel fluorescence images were taken using fluorescence microscopy (Cell Observer, Carl Zeiss Microscopy) under excitation at a wavelength of 470 nm for live cells (emission wavelength 530 nm) and 540 nm for dead cells (emission wavelength 618 nm). After 72 h incubation, the remaining 12 wells of cells were trypsinized, stained and counted under the fluorescence microscope. Cell viability was calculated as the ratio of the number of live cells (green) to the total cell count.

### Statistics and reproducibility

No statistical method was used to predetermine the sample size. No data were excluded from the analyses. Cells and fabricated samples were randomly assigned to the respective groups before operation. The investigators were not blinded to allocation during experiments and outcome assessment.

### Reporting summary

Further information on research design is available in the Nature Portfolio Reporting Summary linked to this article.

## Online content

Any methods, additional references, Nature Portfolio reporting summaries, source data, extended data, supplementary information, acknowledgements, peer review information; details of author contributions and competing interests; and statements of data and code availability are available at 10.1038/s41565-023-01567-0.

### Supplementary information

Supplementary Information Supplementary Figs. 1–17, Tables 1 and 2, Videos 1–8, Texts 1–5 and Refs. 1–29.

Reporting Summary

Supplementary Video 1 Mechanical characterization of the picosprings by the optical trap and finite element analysis simulation. This video shows the characterization of the picospring’s mechanical properties by the optical tweezer and finite element analysis simulation. The characterization is performed by measuring the deflection angle of the cantilever picospring under certain loads based on a classic cantilever beam method. The trapping force of the optical tweezer is obtained according to the velocity of the trapped microbead and the trapping distance (first part). The picospring is deformed by the microbead moved normally to the bending picospring at a negligible velocity by the optical tweezer (second part). The finite element analysis simulation is done by applying certain forces normal to the cantilever picospring (third part).

Supplementary Video 2 Microforcemeter showing visually the energy conversion process in the propulsion force measurement of microswimmers. This video shows the microforcemeter application on the propulsion force measurement of microswimmers. The sperm–motor and the chemical microjet are actuated by the sperm flagellum and O_2_ bubbles, respectively. They are both magnetically guided towards the short action bar of the microforcemeter. Both magnetic microhelices are actuated by a rotating magnetic field at 10 mT/40 Hz. When the microswimmer’s velocity approaches zero, the microforcemeter deflection indicates the propulsion force of the microswimmer.

Supplementary Video 3 Self-closing microgripper performing the tasks of grip, transport and release of a microbead. This video shows the microgripper applications in the capture, transport and release of a microbead as the target object. The microgripper moves toward the microbead with opened fingers under the rotating magnetic actuation field. Once approaching the microbead, the microgripper closes its fingers under the magnetic base field to enclose the microbead inside the bucket. Then it transports the microbead under the rotating magnetic base field. When arriving at the targeted position, the microgripper opens its fingers to release the microbead and swims away under the magnetic actuation field.

Supplementary Video 4 Self-closing microgripper delivering multiple biological objects. This video shows the flexibility of microgripper targets exemplified by the transport of a Hela cell (first part), a mouse sperm (second part) and a protein-based microclot (third part). The microgripper closes its fingers at different angles under different magnetic base fields to grip sensitive objects of different sizes in different shapes. During the transport, the microgripper can be precisely controlled to avoid contact with non-related objects in the environment, ensuring maximum safety. Relying on the gripping-based capturing, the microgripper can adjust the location and orientation of the microobject with high precision, superior to other microrobots without such a transformable end effector.

Supplementary Video 5 Fluorescence live staining showing the safety of the self-closing microgripper during manipulating a HeLa cell. This video shows the transport of a live HeLa cell and the subsequent fluorescence images of the cell after being stained by a live stain. The green fluorescence of the manipulated cell shows that the cell viability was not affected by the manipulation from the microgripper.

Supplementary Video 6 Magnetically actuated oscillation of the microoscillators. This video shows the oscillation of an array of microoscillators under magnetic actuation. From left to right, the microoscillators were fabricated with increasing laser powers and thus have increasing stiffnesses. When actuated by the oscillating magnetic field within an angle of 150° at 10 mT, stiffer microoscillators oscillate with lower amplitudes from left to right.

Supplementary Video 7 Orientation-switching control of the micropenguin under magnetic field based on the stored energy in the picospring. This video shows the locomotion of a transformable micropenguin controlled under an orientation-switching strategy. The micropenguin moves along *+x* direction by closing its flippers at the magnetic actuation field as one stroke. After that, it rotates towards the directions out of (+*z*) or into (*−z*) the page to open its flippers at the magnetic base field. Then it rotates back toward *+x* for another stroke. The micropenguin thus gains a net displacement by periodically switching its orientations between the flippers opening and closing processes. Black arrows show the magnetic field direction. Brown and purple arrows show the changing direction of the magnetic field vector.

Supplementary Video 8 Sequential-motion control of microturtles based on the stored energy in the picospring under the magnetic field. This video shows the locomotion of a transformable microturtle controlled under a sequential-motion strategy. The microturtle consists of four magnetic flippers responsive to external magnetic field and a non-magnetic torso controlled only by elastic force by the zigzag spring linkages. The sequential movements of the left flippers, torso and right flippers actuated by the programmed sequential magnetic fields generate net displacement of the microturtle along its axial axis. The microturtle locomotion does not rely on continuous rolling or rotation, which avoids the friction with the substrate or flow vortex.

Supplementary Data All data for supplementary figures.

### Source data

Source Data Fig. 2 Statistical source data.

Source Data Fig. 3 Statistical source data.

Source Data Fig. 5 Statistical source data.

Source Data Extended Data Fig. 2 Statistical source data.

Source Data Extended Data Fig. 3 Statistical source data.

Source Data Extended Data Fig. 4 Statistical source data.

Source Data Extended Data Fig. 6 Statistical source data.

Source Data Extended Data Fig. 7 Statistical source data.

Source Data Extended Data Fig. 8 Statistical source data.

## Data Availability

All data are available in the main text or supplementary information. Source data are provided with this paper.

## References

[1] Ellison CK (2017). Obstruction of pilus retraction stimulates bacterial surface sensing. Science.

[2] Xu H, Medina‐Sánchez M, Schmidt OG (2020). Magnetic micromotors for multiple motile sperm cells capture, transport, and enzymatic release. Angew. Chem. Int. Ed..

[3] Rudzinska MA (1965). The fine structure and function of the tentacle in *Tokophrya infusionum*. J. Cell Biol..

[4] Sitti M (2018). Miniature soft robots—road to the clinic. Nat. Rev. Mater..

[5] Soto F (2022). Smart materials for microrobots. Chem. Rev..

[6] Palagi S, Fischer P (2018). Bioinspired microrobots. Nat. Rev. Mater..

[7] Vogel V, Sheetz M (2006). Local force and geometry sensing regulate cell functions. Nat. Rev. Mol. Cell Biol..

[8] Cui J (2019). Nanomagnetic encoding of shape-morphing micromachines. Nature.

[9] Dreyfus R (2005). Microscopic artificial swimmers. Nature.

[10] Wang B (2022). Endoscopy-assisted magnetic navigation of biohybrid soft microrobots with rapid endoluminal delivery and imaging. Sci. Robot..

[11] Hu X (2021). Magnetic soft micromachines made of linked microactuator networks. Sci. Adv..

[12] Hu W (2018). Small-scale soft-bodied robot with multimodal locomotion. Nature.

[13] Kim Y (2018). Printing ferromagnetic domains for untethered fast-transforming soft materials. Nature.

[14] Palagi S (2016). Structured light enables biomimetic swimming and versatile locomotion of photoresponsive soft microrobots. Nat. Mater..

[15] Liu D, Wang T, Lu Y (2022). Untethered microrobots for active drug delivery: from rational design to clinical settings. Adv. Healthc. Mater..

[16] Zhang H, Mourran A, Möller M (2017). Dynamic switching of helical microgel ribbons. Nano Lett..

[17] Miskin MZ (2020). Electronically integrated, mass-manufactured, microscopic robots. Nature.

[18] Dabbagh SR (2022). 3D-printed microrobots from design to translation. Nat. Commun..

[19] Baltz JM, Katz DF, Cone RA (1988). Mechanics of sperm–egg interaction at the zona pellucida. Biophys. J..

[20] Xia H (2010). Ferrofluids for fabrication of remotely controllable micro-nanomachines by two-photon polymerization. Adv. Mater..

[21] Zhang S (2019). The optoelectronic microrobot: a versatile toolbox for micromanipulation. Proc. Natl Acad. Sci. USA.

[22] Zeng H (2015). Light-fueled microscopic walkers. Adv. Mater..

[23] Hippler M (2019). Controlling the shape of 3D microstructures by temperature and light. Nat. Commun..

[24] Huang T-Y (2020). Four-dimensional micro-building blocks. Sci. Adv..

[25] Ma Z-C (2020). Femtosecond laser programmed artificial musculoskeletal systems. Nat. Commun..

[26] Bruen D (2020). 3D printed sugar-sensing hydrogels. Macromol..

[27] Ruskowitz ER, DeForest CA (2018). Photoresponsive biomaterials for targeted drug delivery and 4D cell culture. Nat. Rev. Mater..

[28] Maruo S, Nakamura O, Kawata S (1997). Three-dimensional microfabrication with two-photon-absorbed photopolymerization. Opt. Lett..

[29] Yu Y (2017). Bioinspired helical microfibers from microfluidics. Adv. Mater..

[30] Oyunbaatar N-E (2016). Biomechanical characterization of cardiomyocyte using PDMS pillar with microgrooves. Sensors.

[31] Medina-Sánchez M, Schmidt OG (2017). Medical microbots need better imaging and control. Nature.

[32] Lauga E, Powers TR (2009). The hydrodynamics of swimming microorganisms. Rep. Prog. Phys..

[33] Allen MJ (2010). Time-dependent measure of a nanoscale force-pulse driven by the axonemal dynein motors in individual live sperm cells. Nanomed. Nanotechnol. Biol. Med..

[34] Tadir Y (1990). Force generated by human sperm correlated to velocity and determined using a laser generated optical trap. Fertil. Steril..

[35] Miskin MZ (2018). Graphene-based bimorphs for micron-sized, autonomous origami machines. Proc. Natl Acad. Sci. USA.

[36] Jin Q (2020). Untethered single cell grippers for active biopsy. Nano Lett..

[37] Liu QK (2021). Micrometer-sized electrically programmable shape-memory actuators for low-power microrobotics. Sci. Robot..

[38] Goudu SR (2020). Biodegradable untethered magnetic hydrogel milli-grippers. Adv. Funct. Mater..

[39] Shi X (2016). Effects of mechanical stresses on sperm function and fertilization rate in mice. Syst. Biol. Reprod. Med..

[40] Xu H (2020). Sperm micromotors for cargo delivery through flowing blood. ACS Nano.

[41] Caplan LR (1993). Brain embolism, revisited. Neurology.

[42] Wright SL, Kelly FJ (2017). Plastic and human health: a micro issue?. Environ. Sci. Technol..

[43] Schenck JF (1998). MR safety at high magnetic fields. Magn. Reson. Imaging Clin. N. Am..

[44] Schwarz L (2020). A rotating spiral micromotor for noninvasive zygote transfer. Adv. Sci..

[45] Pal A (2021). Exploiting mechanical instabilities in soft robotics: control, sensing, and actuation. Adv. Mater..

[46] Bunea A-I, Glückstad J (2019). Strategies for optical trapping in biological samples: aiming at microrobotic surgeons. Laser Photon. Rev..

[47] Zhou H (2021). Magnetically driven micro and nanorobots. Chem. Rev..

[48] Ng KYB (2018). In vivo oxygen, temperature and pH dynamics in the female reproductive tract and their importance in human conception: a systematic review. Hum. Reprod. Update.

[49] Soto F, Wang J, Ahmed R, Demirci U (2020). Medical micro/nanorobots in precision medicine. Adv. Sci..

[50] Billard A, Kragic D (2019). Trends and challenges in robot manipulation. Science.

[51] Abbott JJ (2007). Modeling magnetic torque and force for controlled manipulation of soft-magnetic bodies. IEEE Trans. Robot..

[52] Striggow F (2020). Sperm-driven micromotors moving in oviduct fluid and viscoelastic media. Small.

[53] Mayer F (2019). Multimaterial 3D laser microprinting using an integrated microfluidic system. Sci. Adv..

[54] Purcell EM (1977). Life at low Reynolds number. Am. J. Phys..

