# Author Correction: 3D nanofabricated soft microrobots with super-compliant picoforce springs as onboard sensors and actuators

**DOI:** 10.1038/s41565-024-01647-9

**Published:** 2024-03-18

**Authors:** Haifeng Xu, Song Wu, Yuan Liu, Xiaopu Wang, Artem K. Efremov, Lei Wang, John S. McCaskill, Mariana Medina-Sánchez, Oliver G. Schmidt

**Affiliations:** 1 Shenzhen Institute of Advanced Technology (SIAT), Chinese Academy of Sciences, Shenzhen, China; 2 Leibniz Institute for Solid State and Materials Research Dresden (Leibniz IFW Dresden), Dresden, Germany; 3 Shenzhen Institute of Artificial Intelligence and Robotics for Society, Shenzhen, China; 4 Shenzhen Bay Laboratory, Shenzhen, China; 5 Research Center for Materials, Architectures and Integration of Nanomembranes (MAIN), Chemnitz University of Technology, Chemnitz, Germany; 6 Chair of Micro- and NanoSystems, Center for Molecular Bioengineering (B CUBE), Dresden University of Technology, Dresden, Germany

**Keywords:** Mechanical engineering, Biosensors, Materials for devices, Design, synthesis and processing

Correction to: *Nature Nanotechnology* 10.1038/s41565-023-01567-0, published online 3 January 2024.

In the version of the article initially published, in Fig. 4c, the units of the approach and transport velocities were erroneously listed as μm/min instead of μm s^–1^. This has now been corrected in the HTML and PDF versions of the article.

