# Supplementary material for: 3D nanofabricated soft microrobots with super-compliant picoforce springs as onboard sensors and actuators
Source: Nat Nanotechnol. 2024 Jan 3;19(4):494–503. doi: 10.1038/s41565-023-01567-0 (PMC11026159; doi:10.1038/s41565-023-01567-0)
Supplement: Supplementary file 1 — Supplementary Figs. 1–17, Tables 1 and 2, Videos 1–8, Texts 1–5 and Refs. 1–29. [file 41565_2023_1567_MOESM1_ESM.pdf]

nature nanotechnology

Article

https://doi.org/10.1038/s41565-023-01567-0

3D nanofabricated soft microrobots with super-compliant picoforce springs as onboard sensors and actuators

In the format provided by the authors and unedited

Contents

Supplementary Figs. 1-17 2

Supplementary Tables S1-S2 16

Supplementary Videos 1 to 8 18

Supplementary Text Sections S1-S5 21

References: 29

Supplementary Figs. 1-17

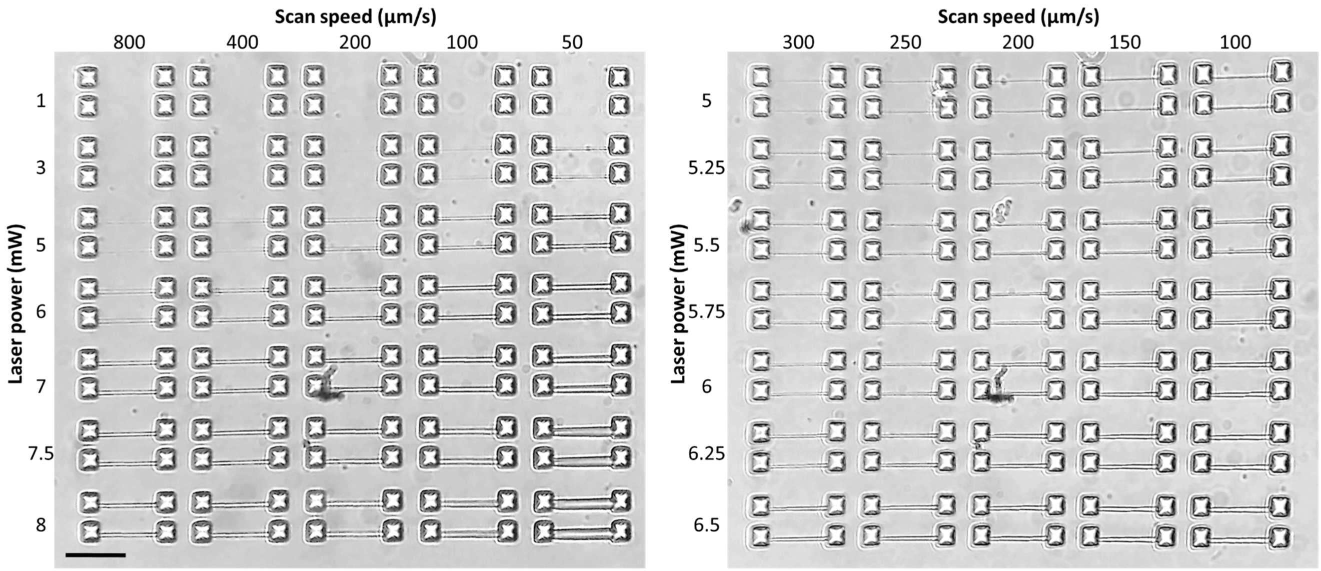

Supplementary Fig. 1. Influence of laser parameters on the structural quality of the fabricated picosprings after media change. The beam-shape picosprings are replicated twice, for each laser power and scan speed, to show the fabrication stability. The rigid platforms where the picosprings are fixed are fabricated at laser power 25 mW and scan speed 400 μm/s. The right-hand panel shows the most useful central portion of the parameter space with higher parameter resolution. Scale bar: 20 μm.

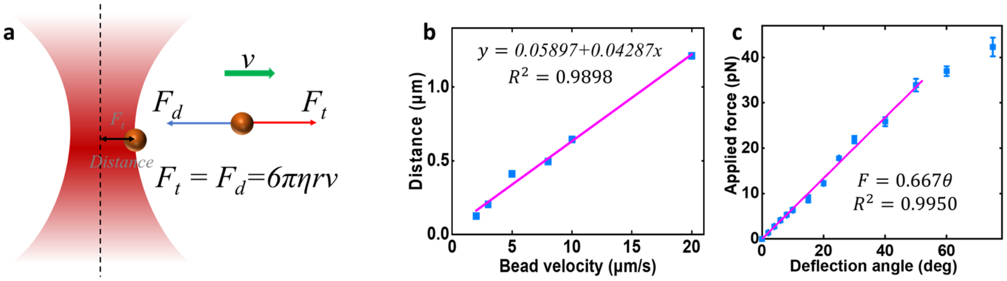

Supplementary Fig. 2. Calibration of the trapping force of the optical tweezer system. (A) Schematic showing the calibration principle of the optical trap force according to the radius and velocity of a microbead. (B) Calibration curve of the optical trap constant. (C) Calibration curve by an optical tweezer (n = 3 measurements, mean ± s.d.). Red line indicates the linear relationship between the elastic force and deflection angle within 50°.

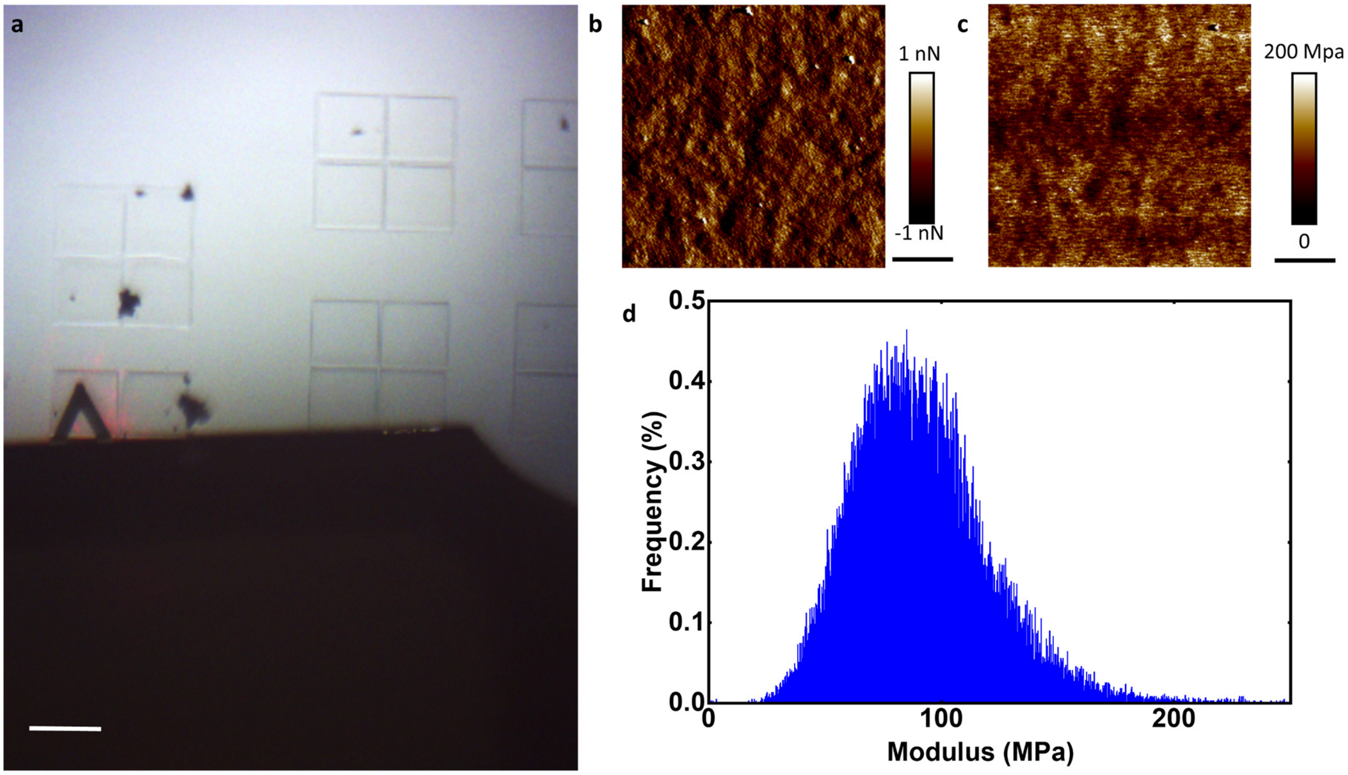

Supplementary Fig. 3. Mechanical characterization of the rigid part of the picospring material fabricated at 25 mW. (a) Optical image of the characterization process by atom force microscopy (AFM). The modulus of the material is averaged over four samples, giving 98.471±10.332 Mpa (mean±s.d.). Scale bar: 100 µm. (b) Picoforce error. Scale bar: 1 µm. (c) DMT (Derjaguin-Muller-Toporov) modulus. Scale bar: 1 µm. (d) Modulus distribution of one sample.

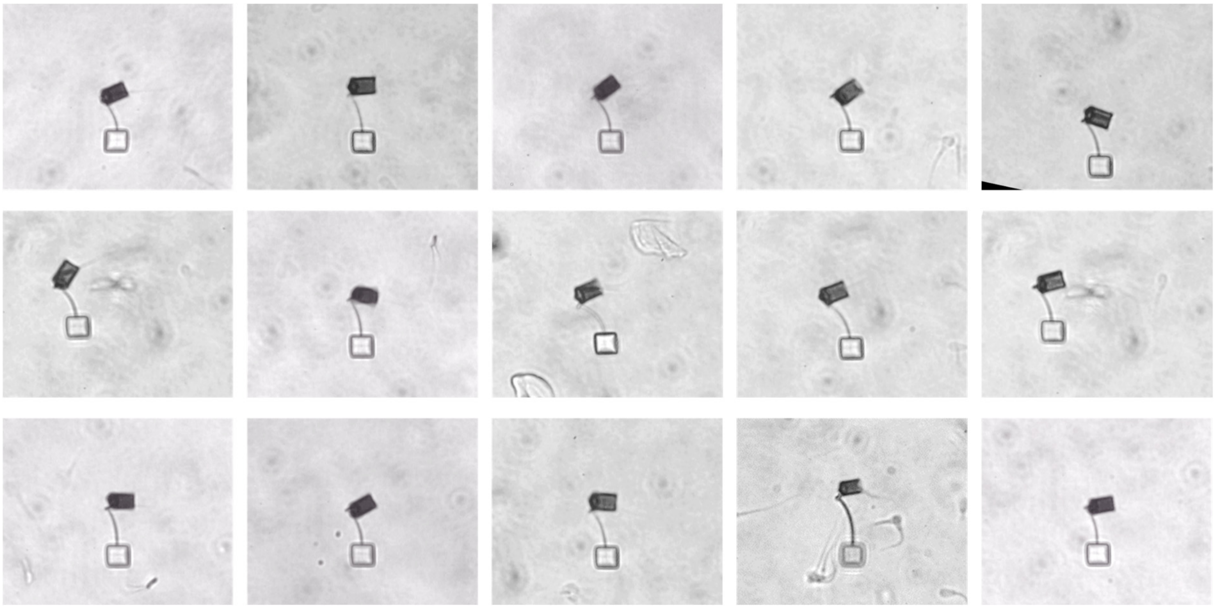

Supplementary Fig. 4. Microforcemeter deformation under the propulsion force for 15 samples of the 1-tube sperm-motors 37 °C.

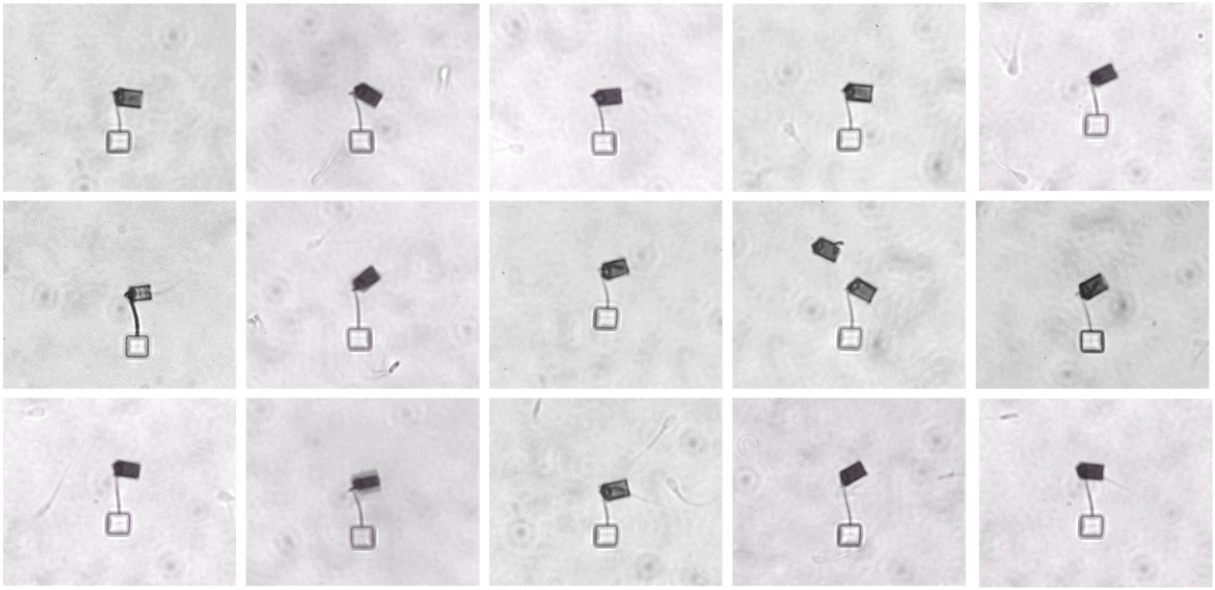

Supplementary Fig. 5. Microforcemeter deformation under the propulsion force for 15 samples of the 1-tube sperm-motors at 25 °C.

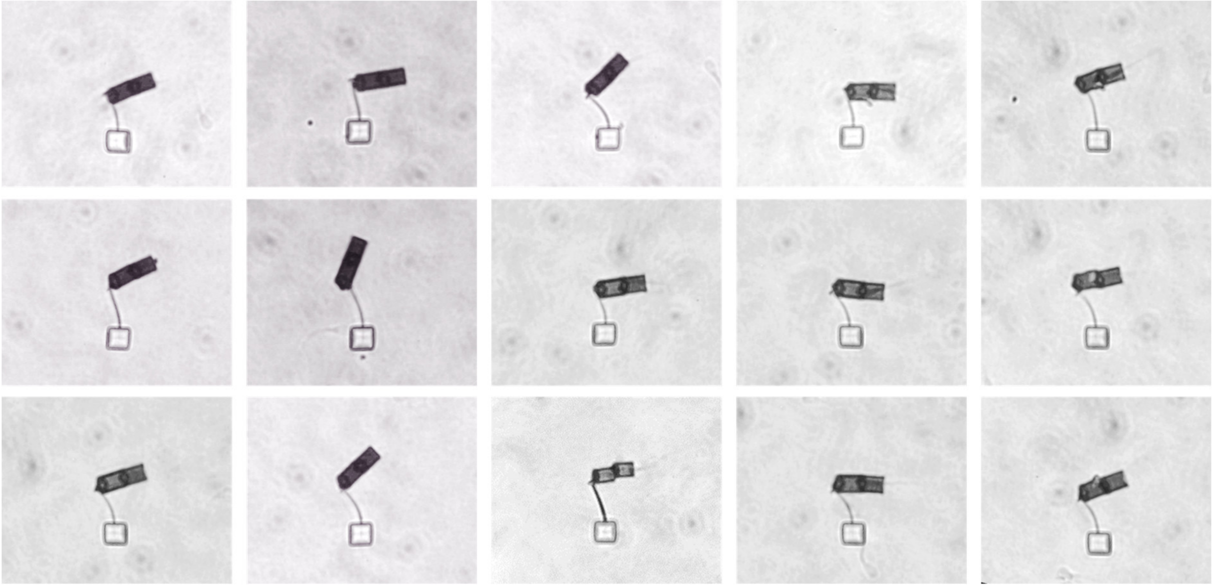

Supplementary Fig. 6. Microforcemeter deformation under the propulsion force for 15 samples of the 2-tube sperm-motors.

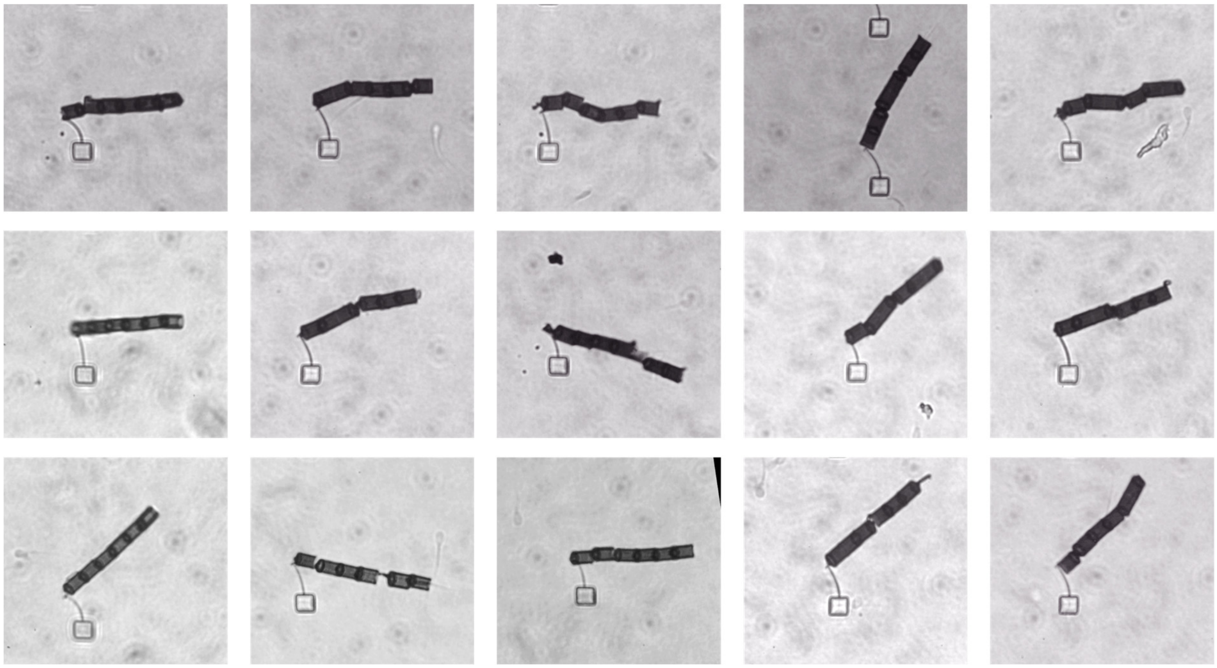

Supplementary Fig. 7. Microforcemeter deformation under the propulsion force for 15 samples of the 6-tube sperm-motors.

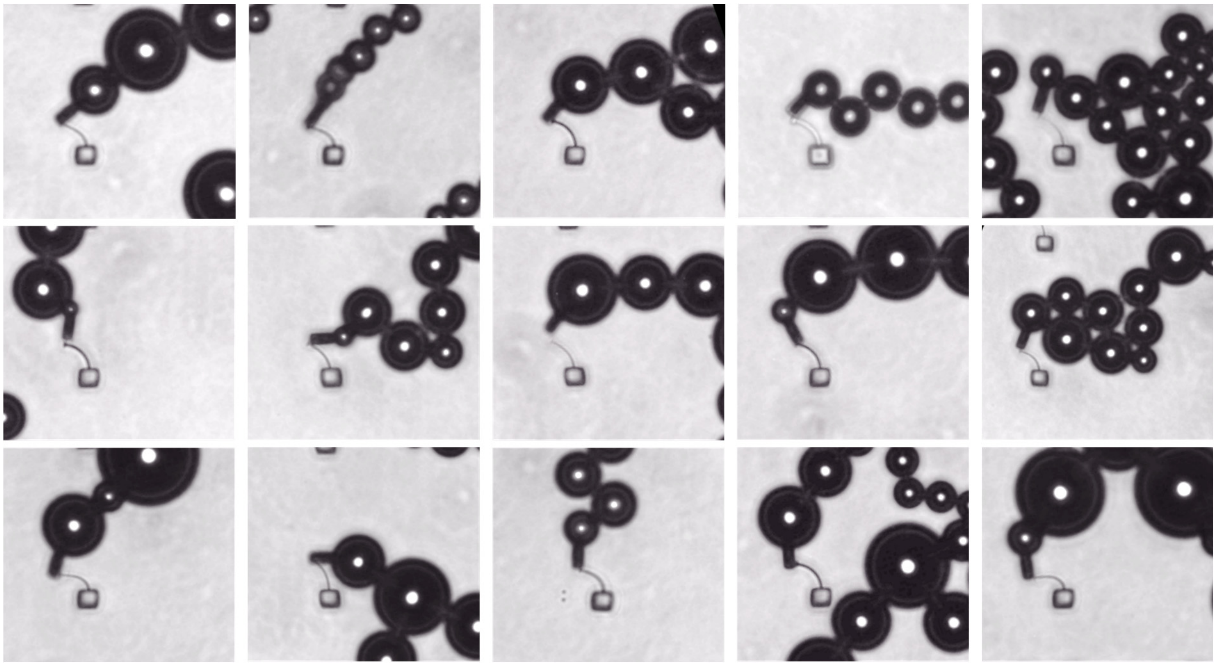

Supplementary Fig. 8. Microforcemeter deformation under the propulsion force for 15 samples of the chemical microjets.

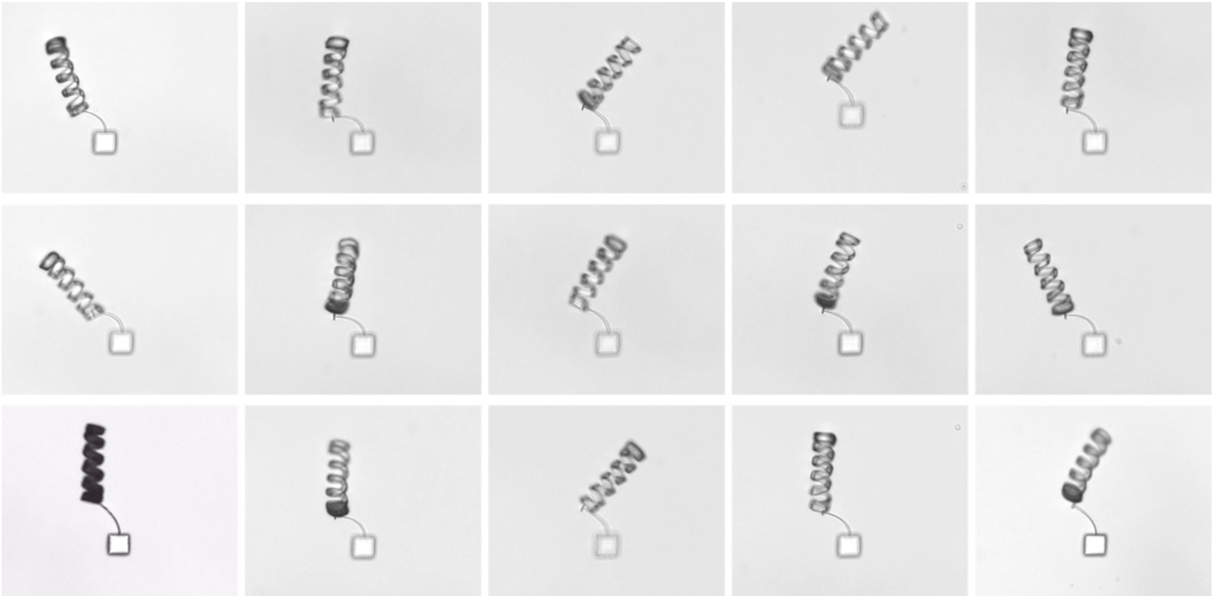

Supplementary Fig. 9. Microforcemeter deformation under the propulsion force for 15 samples of the long microhelix.

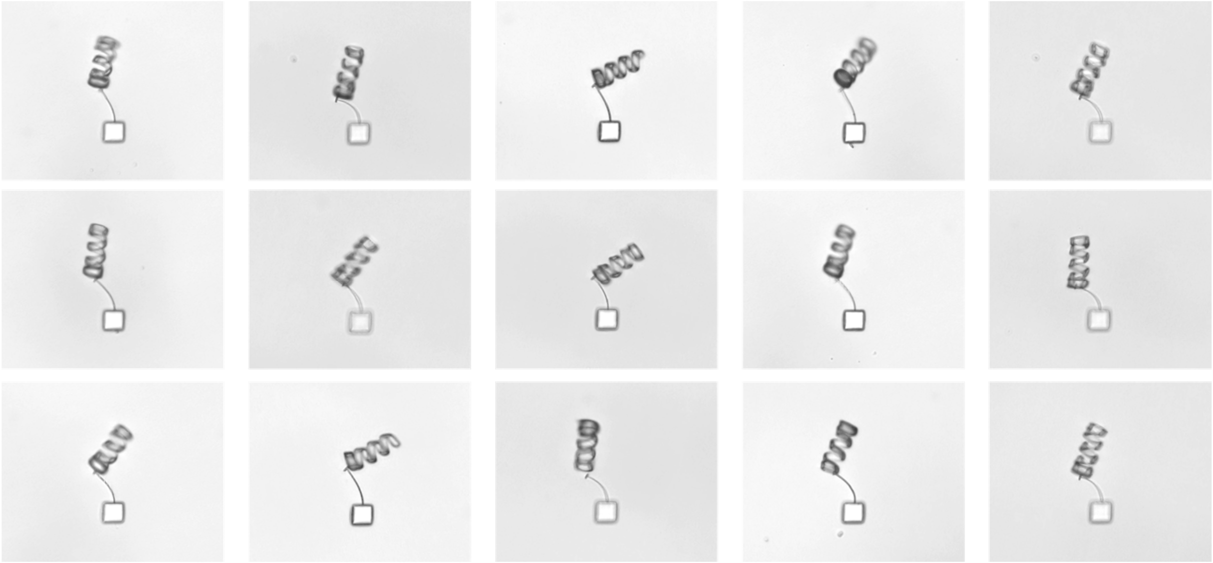

Supplementary Fig. 10. Microforcemeter deformation under the propulsion force for 15 samples of the short microhelix.

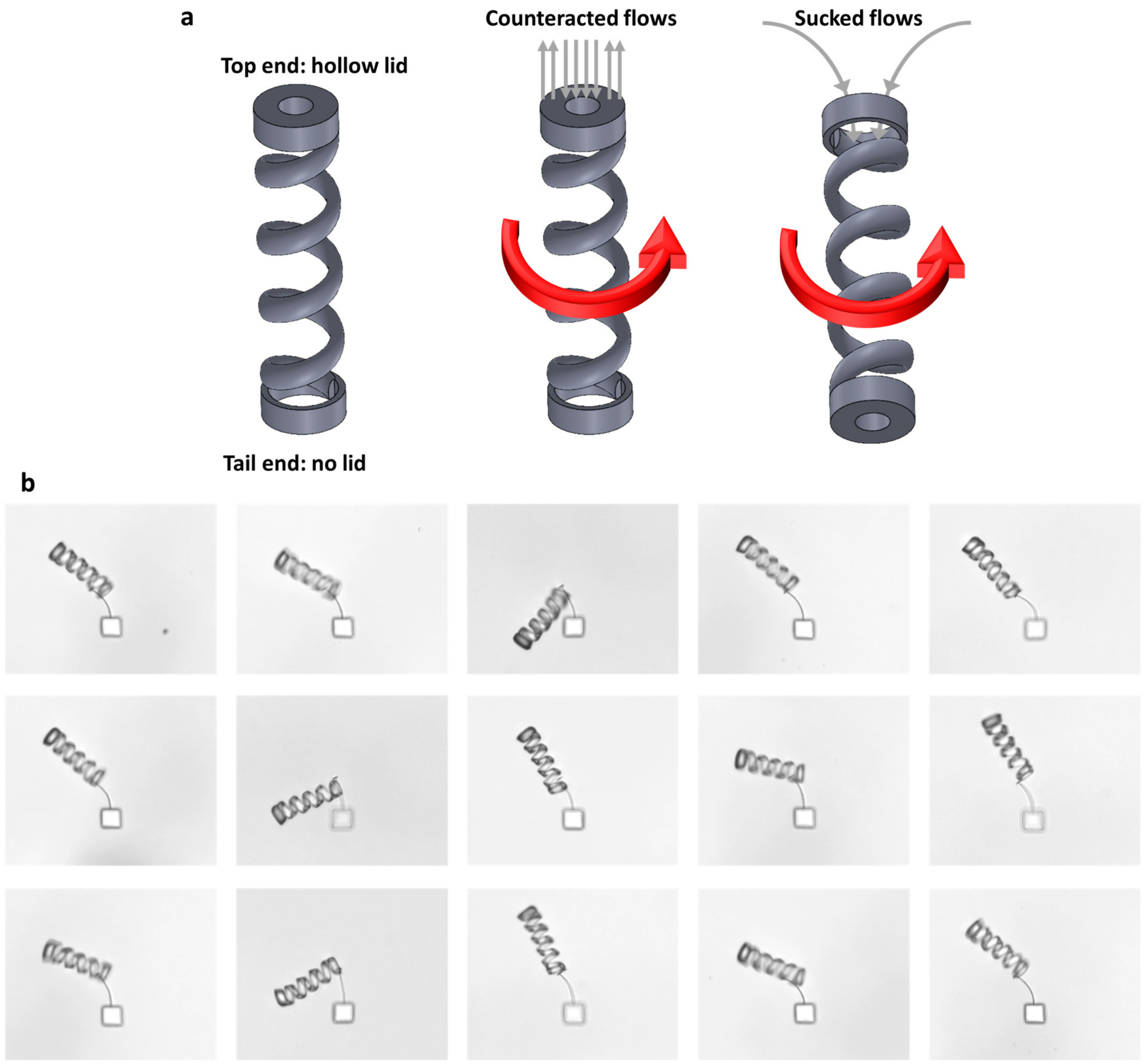

Supplementary Fig. 11. Microforcemeter deformation under the sucking force for 15 samples of the long microhelix. (a) Microhelix generating different flows when swimming toward opposite directions. (b) Measurement of 15 samples.

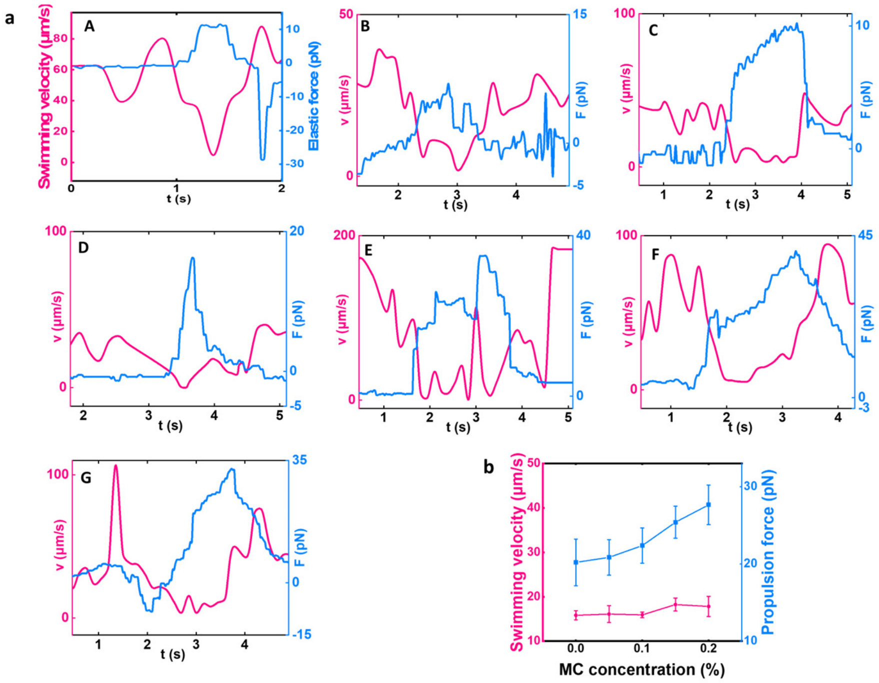

Supplementary Fig. 12. Measurement results of the propulsion forces and swimming velocities of different microswimmers. (a) Swimming velocities of sperm-motors and the related elastic forces of the deformed cantilevers over time. (A) 1-tube sperm-motor; (B) 1-tube sperm-motor at 25 °C; (C) 2-tube sperm-motor; (D) 6-tube sperm-motor. (E) chemical microjet; (F) long microhelix; (G) short microhelix. (b) Viscosity dependent propulsion of the long microhelix swimming in methylcellulose solutions at different concentrations (n = 3 measurements, mean ± s.d.).

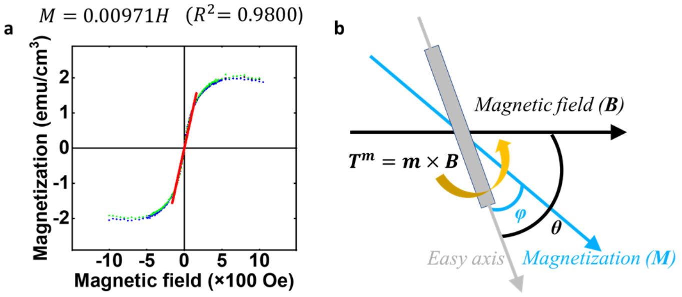

Supplementary Fig. 13. Magnetic properties of the elastic system responsive to the external magnetic field. (a) SQUID measurement of the elastic system embedded with magnetic particles by. Red line indicates the linear fit within ± 160 Oe. (b) Schematic illustration of the magnetic torque applied on a soft-magnetic beam due to the misalignment of the easy axis and magnetization axis. The magnetization direction of the beam is in between its easy axis and the external magnetic field direction due to the geometrical anisotropy of the structure.

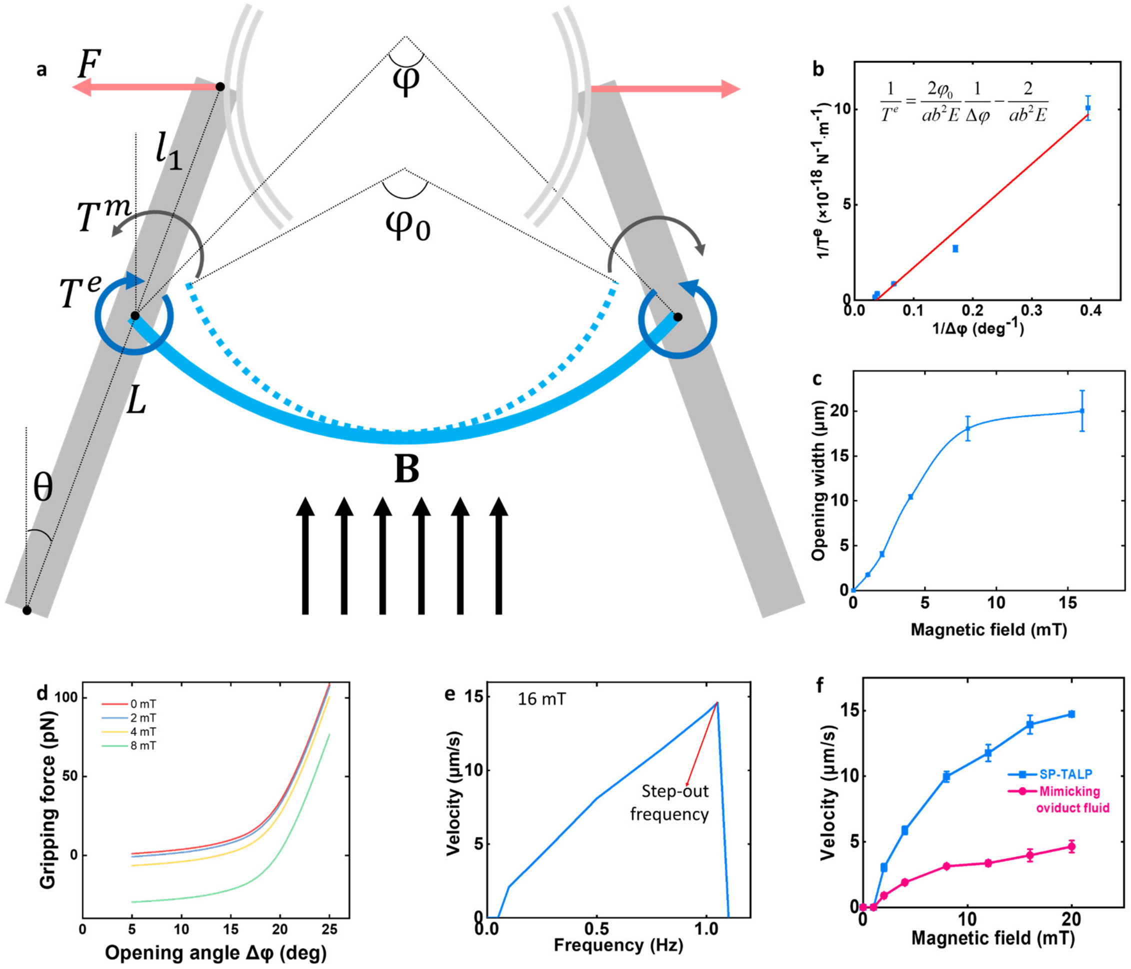

Supplementary Fig. 14. Dynamical behaviour of the microgripper. (a) Schematic of the gripping force based on a pure bending model of the arc picosprings. (b) Linear fitting of the elastic torque equation ($R^2 = 0.9837$). Mean ± s.d. represents the measurement of three individual microgrippers. (c) Microgripper opening width relative to the applied magnetic field (n = 3 microgrippers for each group, mean ± s.d.). (d) Gripping force depending on the opening angle under different magnetic fields. (e) Increasing locomotion velocity with increasing rotation frequency of the magnetic field at 16 mT. For a fixed magnetic field intensity, the microgripper velocity increases with its rotation frequency before the step-out frequency. (f) Increasing locomotion velocity with increasing magnetic field at the step-out frequencies (n = 3 microgrippers for each group, mean ± s.d.). At relevant step-out frequencies, the transport velocity is positively correlated with the applied magnetic field.

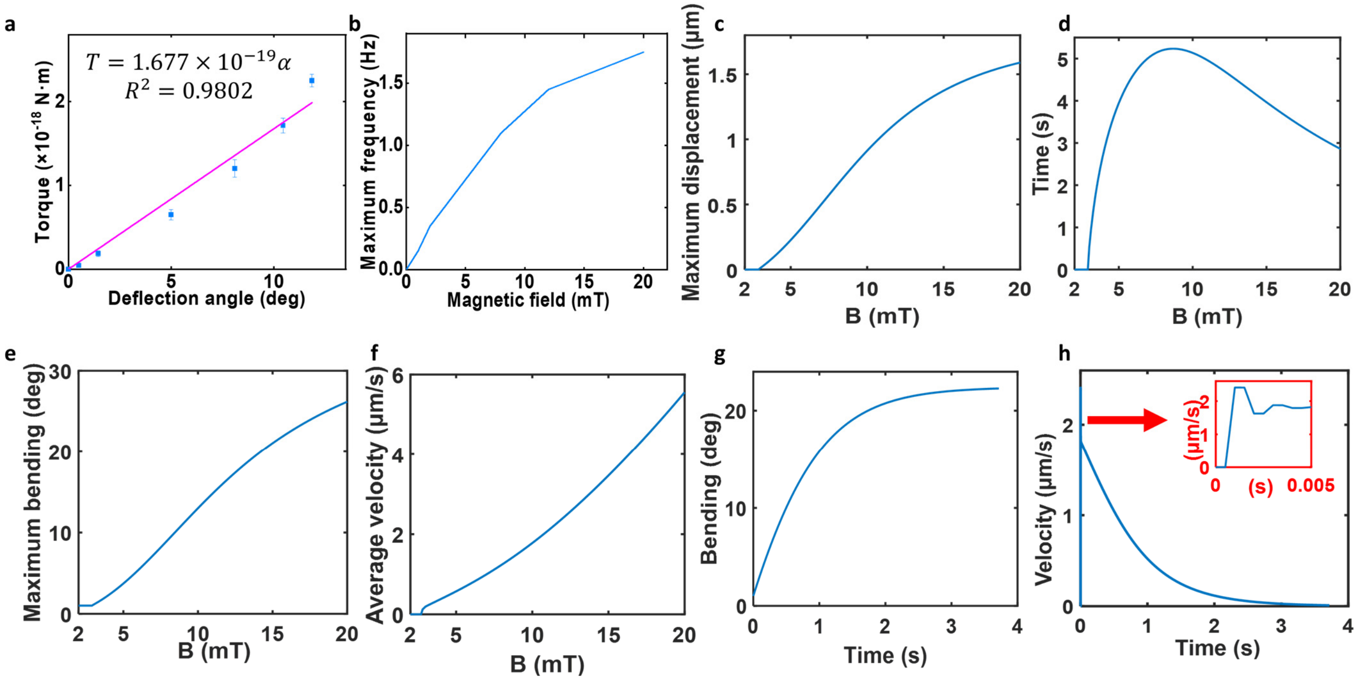

Supplementary Fig. 15. Dynamic analysis of the soft micropenguin. (a) The deflection angle of the picospring linkage with respect to the magnetic torque of the flipper. The deflection angle is defined as half of the rotation angle of the flipper. Bending stiffness of the picospring is approximately determined by the slope of the fitting curve (n = 3 micrograppers for each group, mean ± s.d.). (b) Maximum rotation frequency (step-out frequency) of the micropenguin according to a rotation test under different magnetic fields. (v) Theoretical micropenguin displacement, (d) stroke time, (e) maximum bending and (f) average velocity as functions of the magnetic actuation field. (g) Theoretical deflection angle and (h) instantaneous velocity over time during one stroke under a certain magnetic actuation field of 16 mT. Inset of (e) shows the velocity profile over the first 5 ms, indicating a very short stabilizing time at low Reynolds number.

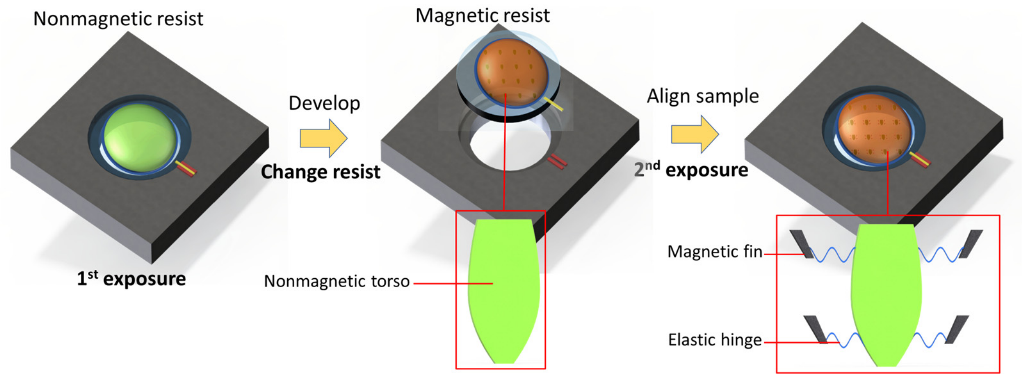

Supplementary Fig. 16. Schematic illustration of the separate exposures of the magnetic and nonmagnetic parts of a partially magnetic microrobot.

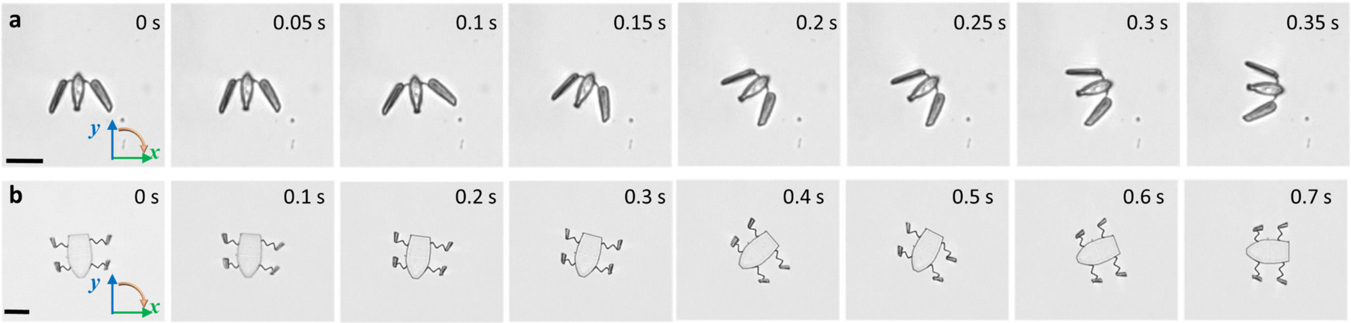

Supplementary Fig. 17. Steering of the transformable microrobots. (a) Micropenguin rotating by 90° within 0.35 s. (b) Microturtle turning by 90 ° within 0.7 s. Brown curved arrows indicate the turning direction of the microrobots orientations from +y to +x. The micropenguin can rotate as a whole under the magnetic field while the microturtle uses its magnetic flippers as paddles to rotate. Scale bars: 30 μm.

Supplementary Tables S1-S2

Table 1. Parameters in the theoretic model of the micropenguin.

Parameter	Value	Meaning
L_1	12 μm	Length of the left fin
L_2	12 μm	Length of the right fin
L_3	16 μm	Length of the torso
L_s	5 μm	Length of the spring connecting rod
w_1	2 μm	Width of the left fin
w_2	2 μm	Width of the right fin
w_3	4 μm	Width of the torso
h	4 μm	Height of the micropenguin
k^e	1.677×10^{-19} N·m/deg	Bending stiffness of the spring
η	1 mPa·s	Fluid viscosity
χ	0.1220	Magnetic susceptibility
θ_0	37.3 deg	Initial orientation angle of the fin
L_c		Chord length of the spring
θ		Orientation angle of the fin
$\dot{\theta}$		Angular speed of the fin
$\dot{x}$		Locomotion velocity of the torso

Table 2. Locomotion velocities of state-of-the-art artificial microrobots.

**Types of microswimmers** *Artificial microrobots*	**Driving mechanism**	**Dimension**	**Speed (BL/s)**	**Speed**	**Reference**
Voltage-controllable electrochemical actuators	Bending in response to electrochemically driven adsorption	70 μm	~0.43	30 μm/s	1
Surface-slipping mobile microrobots	Acoustic radiation force and magnetic navigation	25 μm	0.11	~2250 μm/s	2
RoboWorm	Worm-like crawling based on optogenetic excitation	~450 μm	0.11	50 μm/s	3
Sperm-templated soft magnetic microrobots	Magnetic rotation	~34 μm	0.2	6.8 ± 4.1 μm/s	4
Adaptive shape-morphing microrobots	Magnetic force	~83 μm	~0.72	60 μm/s	5
Microrobots driven by artificial muscles	Artificial muscles	~800 μm	~0.0011-0.0013	0.88~1.05 μm/s	6
Helix microrobots	Magnetic rotation	25 μm	1.78~3	44.5±2.4~4.9 ± 4.1 μm/s	7
Self-Walking gel	Autonomous swelling–deswelling oscillation	6 mm	0.0005	170 μm/min	8
Burr-like porous spherical microrobot	Magnetic force based on the magnetic field gradient	~80 μm	~18.75	~1500 μm/s	9
Nanotweezers	Magnetic rotation	~2 μm	0.35	0.7 μm/s	10
Photoactive liquid-crystal microrobots	Travelling-wave deformations	~1.23 mm	~0.0023	2.8 μm/s	11
Magnetic actuated pH-responsive hydrogel based soft micro-robot	Magnetic force under a gradient field	1.8 mm	~0.33	600 μm/s	12
Flexible nanowire motors	Magnetic rotation	5.8 μm	3.62	21 μm/s	13

Light-Fueled Microscopic Walkers	Light induced stress	60 µm	~6.33	380 µm/s	14
**Types of microswimmers**<br>*Natural microswimmers*	**Driving mechanism**	**Dimension**	**Speed (BL/s)**	**Speed**	**Reference**
Paratarsotomus macropalpis	Alternating tetrapod gait	0.7 mm	274.86	192.4±2.1 mm/s	15
Human sperm	Flagellar propulsion	55-65 µm	1.07~1.27	70±5 µm/s	16
Paramecia	Rhythmical cilia beating	0.32 mm	0.21~5.81	0.71±.08~1.86 ± 0.16 mm/s	17
Macrophage	Ameboid movement/ mesenchymal migration mode	21 µm	0.0009~0.0018	1.13±0.16~ 2.37 ± 0.13 µm/min	18
Escherichia coli	Flagellar propulsion	1~2 µm	0.14~0.28	17±5 µm/min	19

Supplementary Videos 1 to 8

Video 1. Mechanical characterization of the picosprings by the optical trap and FEA simulation.

This video shows the characterization of the picospring's mechanical properties by the optical tweezer and FEA simulation. The characterization is performed by measuring the deflection angle of the cantilever picospring under certain loads based on a classic cantilever beam method. The trapping force of the optical tweezer is obtained according to the velocity of the trapped microbead and the trapping distance (first part). The picospring is deformed by the microbead moved normally to the bending picospring at a negligible velocity by the optical tweezer (second part). The FEA simulation is done by applying certain forces normal to the cantilever picospring (third part).

Video 2. Microforcemeter displaying visually the energy conversion process in the propulsion force measurement of microswimmers.

This video shows the microforcemeter application on the propulsion force measurement of microswimmers. The sperm-motor and the chemical microjet are actuated by the sperm flagellum

and O_2 bubbles, respectively. They are both magnetically guided toward the short action bar of the microforcemeter. Both magnetic microhelices are actuated by a rotating magnetic field at 10 mT/40 Hz. When the microswimmer's velocity approaches 0, the microforcemeter deflection indicates the propulsion force of the microswimmer.

Video 3. Self-closing microgripper performing the tasks of grip, transport and release of a microbead.

This video shows the microgripper applications in the capture, transport and release of a microbead as the target object. The microgripper moves toward the microbead with opened fingers under the rotating magnetic actuation field. Once approaching the microbead, the microgripper closes its fingers under the magnetic base field to enclose the microbead inside the bucket. Then it transports the microbead under the rotating magnetic base field. When arriving at the targeted position, the microgripper opens its fingers to release the microbead and swims away under the magnetic actuation field.

Video 4. Self-closing microgripper delivering multiple biological objects.

This video shows the flexibility of microgripper targets exemplified by the transport of a Hela cell (first part), a mouse sperm (second part) and a protein-based microclot (third part). The microgripper closes its fingers at different angles under different magnetic base fields to grip sensitive objects of different sizes in different shapes. During the transport, the microgripper can be precisely controlled to avoid contact with nonrelated objects in the environment, ensuring maximum safety. Relying on the gripping-based capturing, the microgripper can adjust the location and orientation of the microobject with high precision, superior to other microrobots without such a transformable end effector.

Video 5. Fluorescence live staining showing the safety of the self-closing microgripper during manipulating a HeLa cell.

This video shows the transport of a live HeLa cell and the subsequent fluorescence images of the cell after being stained by a live stain. The green fluorescence of the manipulated cell shows that the cell viability was not affected by the manipulation from the microgripper.

Video 6. Magnetically actuated oscillation of the microoscillators.

This video shows the oscillation of an array of microoscillators under magnetic actuation. From left to right, the microoscillators were fabricated with increasing laser powers and thus have increasing stiffnesses. When actuated by the oscillating magnetic field within an angle of 150° at 10 mT, stiffer microoscillators oscillate with lower amplitudes from left to right.

Video 7. Orientation-switching control of the micropenguin under magnetic field based on the

stored energy in the picospring.

This video shows the locomotion of a transformable micropenguin controlled under an orientation-switching strategy. The micropenguin moves along $+x$ direction by closing its flippers at the magnetic actuation field as one stroke. After that, it rotates toward the directions out of ($+z$) or into ($-z$) the page to open its flippers at the magnetic base field. Then it rotates back toward $+x$ for another stroke. The micropenguin thus gains a net displacement by periodically switching its orientations between the flippers opening and closing processes. Black arrows show the magnetic field direction. Brown and purple arrows show the changing direction of the magnetic field vector.

Video 8. Sequential-motion control of microturtles based on the stored energy in the picospring under the magnetic field.

This video shows the locomotion of a transformable microturtle controlled under a sequential-motion strategy. The microturtle consists of four magnetic flippers responsive to external magnetic field and a nonmagnetic torso controlled only by elastic force by the zigzag spring linkages. The sequential movements of the left flippers, torso and right flippers actuated by the programmed sequential magnetic fields generate net displacement of the microturtle along its axial axis. The microturtle locomotion does not rely on continuous rolling or rotation, which avoids the friction with the substrate or flow vortex.

Supplementary Text Sections S1-S5

S1. Piconewton-scale compliance of the micrometer-sized structure

S1.1. Fabrication

Polymeric materials containing N or O atoms swell in water under the action of hydrogen bonds. Consequently, elastic microstructures are easily deformed by disordered oscillation under the high stress at the interface between water and the organic developer of high fluidity during the solvent changing process. This water-induced damaging leads to the failure of many elastic microstructures in bio-environments. To avoid this, intermediate solutions containing a thickener at gradient concentrations are used throughout the media change process until the surrounding environment is totally replaced by water or cell media (**Extended Data Fig. 1a**). The thickener enhances the solution viscosity, thus protecting the microstructure by inhibiting the microstructure oscillation (**Extended Data Fig. 1b**). Although the media change could be done manually, a more efficient way is to use a microfluidic pump with multiple channels. Programming the introduction of all the changing solutions can save much effort.

The picospring stiffness and quality is dependent on the fabrication laser parameters. **Supplementary Fig. 1** shows the optical images of beam-shaped picosprings under the laser power and scan speed test. The picosprings were fabricated 5 μm high away from the substrate to maximumly simulate the application scenario. In this case, the picosprings were not stabilized by the substrate and showed their real structural quality better. Stiffer picosprings with better quality were obtained at higher laser powers and lower scan speed. Making good use of the laser power during lithography, various functional elastic devices can be fabricated out of PEGDA-UA oligomers with programmable elasticity, such as a microforcemeter based on a cantilever picospring, a micropenguin based on coil springs, a microturtle based on zig-zag springs and a microgripper based on arc springs (**Extended Data Fig. 1c**). Considering the fabrication efficiency, we finally set the scan speed as 200 μm/s. Considering the applicable compliance at the pN scale, we set the laser power of the cantilever picospring for the microforcemeter as 5.5 mW, the zig-zag, coil and arc springs for microrobots and microgripper as 6.0 mW and all rigid parts as 25 mW.

The picospring provides a feasible technique to fabricate extremely small robots. By exploring different actuation mechanisms and optimizing magnetic configurations, we can continue to push the boundaries of miniaturization in the microrobotics realm. While softer picosprings can indeed deform faster and to a larger extent, they also require more time for recovery. In order to strike a balance between deformation speed and recovery time, we have opted to fabricate picosprings at a relatively high laser power (6.0 mW) for our microrobots, within the limits of our maximum magnetic field capabilities. For cases where less deflection is required, or enhanced magnetic response can be achieved, smaller elastic micromachines can be custom-designed accordingly, with a downscaling limit determined by the dimension of the picospring with a cross-section of ca. 0.5 μm^2.

S1.2. Elasticity characterization

As shown in **Supplementary Fig. 3**, the mechanical characterization the rigid parts of the material was determined by a Dimension Icon (Bruker, USA) AFM in PeakForce Quantitative Nano-Mechanics mode. 100×100×5 μm^3 platforms were fabricated with certain laser powers as samples for characterization. A Bruker probe SNL-B with a nominal spring constant of 0.12 N/m and tip radius of 2 nm was used. The measurement was done in SP-TALP under hydrated conditions in a 60 mm petri dish. An applied load of up to 1 nN was applied. The Young's modulus E was calculated from three measurements by fitting the force curves: $F = \frac{4E\delta^{3/2}\sqrt{R}}{3(1-\nu^2)}$, where F is the applied force, R the tip radius, δ the indentation distance and ν the Poisson ratio, taken as 0.499 for a general UAO resin[20]. Before measurement, the deflection sensitivity was calibrated on a clean glass surface and the spring constant was calibrated as 0.0917 N/m by the thermal noise method, using the built-in software.

Cantilever picosprings were fabricated for characterizing the mechanical property of the elastic material based on a classic cantilever beam method. An optical tweezer or any other mechanical system with low-force resolution can easily characterize the cantilever picospring. In an optical tweezer system produced by a focused Gaussian beam, when the laser focus strikes a microbead, the trapping kinetics is described by a damped oscillator as (*57*) $m\ddot{x} + a\dot{x} + kx = 0$, where x is the distance between the particle and the laser focus, m is the bead mass, a represents the resistance constant and k represents the trapping force constant of the approximate harmonic potential (**Supplementary Fig. 2a**). At low Reynolds number condition, we have $6\pi\eta r\dot{x} + ax = 0$ *i.e.* $x = (6\pi\eta r/a)v$, according to the Stokes-Einstein equation, where η, r, and v represent the solution viscosity, the bead radius (5 μm in this study), and the bead velocity, respectively. The optical tweezer can then be characterized by linearly fitting x and v, providing the accurate number of the trapping force (**Supplementary Fig. 2b**). The microbead moves parallel to the short bar on the characterization cantilever, *i.e.* towards the normal direction of the cantilever. The applied force can then be calculated according to the trapping force constant and the relative distance between the microbead and the trap coordinates. The sine of the deflection angle is directly defined as the ratio of the lateral displacement of the cantilever's free end to the distance from the current position of its free end to its fixed end. The elastic property of the picospring is decided by the laser power during the cantilever fabrication. This work focuses on the cantilever at 5.5 mW which fits the sperm-motor measurement best. The result at small deflection reveals a linear relationship between the deflection angle and the applied force, of which the determination coefficient R^2 is 0.9950. In terms of the large deflection of an isotropic elastic solid, the relation between its displacement vector **u** and load $\mathbf{f}_v$ (load force per unit volume) can be summarized as Navier's equations:

$$\frac{E}{2(1+\nu)}\left(\frac{1}{(1-2\nu)}\nabla(\nabla\cdot\mathbf{u}) + \nabla^2\mathbf{u}\right) + \mathbf{f}_\mathbf{V} = \rho\frac{\partial^2\mathbf{u}}{\partial t^2},$$

where E and ν represent the elastic modulus and poison ratio of the solid continuum. A finite element analysis (FEA) method can solve the equation after determining essential parameters. The geometry parameters were obtained by high-resolution confocal laser scanning microscopy (CLSM) as shown in **Fig. 1C**. Then a parametric sweep of the applied force is implemented on the structure by FEA when using the young's modulus obtained by the optical trap (**Extended Data Fig. 3**). The variance between the simulated results and the experimental results gets larger at high deflection level (>50°), which can be attributed to the geometry error during fabrication and the hyperelasticity of the material at high loads.

The microforcemeter has a similar structure and compliance as the characterization cantilever

picospring. Simulated curves of the microforcemeter and the characterization picospring coincides well with each other (**Extended Data Fig. 3b-e**). The calibrated microforcemeter can be used to measure pN-scale propulsion forces of microswimmers as shown in **Fig. 3** and **Supplementary Fig. 4-12**.

S2. Magnetic torque on the elastomeric material embedded with superparamagnetic nanoparticles.

In principle, magnetic actuation based on shape morphing requires the microrobot to be able to recover its shape change induced by the magnetic field or other mechanisms. This was demonstrated previously by using pre-magnetized single-domain nanomagnets [21] and linked magnetic microparticles [22,23] at the micrometer scale. Larger robots with simple geometries have also been developed with small magnets [24] and soft materials containing aligned magnetic particles [25]. However, none of these actuation strategies can be combined with a fully integrated 3D fabrication strategy, which restricts the design freedom and locomotion modes of the microrobots.

The material magnetization was characterized by a superconducting quantum interference device (SQUID) magnetometer (**Supplementary Fig. 5a**). The sample was fabricated as an array of microscale cuboids with a similar size to the magnetic parts of the present micromachines. Details of parameter characterization can be found in Methods Section. The magnetization of the MNP embedded material is highly linear in response to the applied magnetic field below 16 mT with a magnetic susceptibility of 0.1220.

The driving power of a magnetic material under the magnetic field typically relies on either the dipole-dipole interaction or the magnetic torque. The former, working in a gradient magnetic field, needs a high magnetic field source to ensure an adequate field at the working position. The inhomogeneity of the field distribution also presents barriers for microrobot control. The torque-based actuation can be operated in uniform magnetic field. **Supplementary Fig. 5b** shows the action of the magnetic field on a soft magnetic beam with geometrical anisotropy. A magnetization direction is created for the magnetic beam between its geometrical easy axis and the magnetic field direction. The magnetic torque corresponding to the angle between the magnetization direction the magnetic field direction (θ-φ) drives the beam to rotate towards the magnetic field direction. The magnetic torque on the magnetic beam satisfies $\mathbf{T} = \mathbf{m} \times \mathbf{B}$, *i.e.* $T_1^m = \mathrm{mBsin}(\theta - \phi)$, where θ and ϕ are the angles from the magnetic field to the easy magnetic axis of the segment and the magnetization direction, respectively. Here $\phi = \theta$, when the magnetic field is parallel to the axial direction of the torso (+*x*). As shown in **Supplementary Fig. 5b**, the magnetic beam as a soft magnetic material satisfies

$$\begin{cases} T_1^m = \dfrac{\chi V B^2 sin(\theta - \phi)}{\mu} \sqrt{(\dfrac{cos\theta}{1+\chi n_L})^2 + (\dfrac{sin\theta}{1+\chi n_w})^2} \\ \phi = arctan(tan\,\theta \cdot \dfrac{1+\chi n_L}{1+\chi n_w}) \end{cases}, \quad (1)$$

where V is the bulk volume of the beam, and χ and μ represent the magnetic susceptibility and the magnetic permeability of water. n_L and n_w are the demagnetization factors in the axial and lateral directions, respectively satisfying

$$\begin{cases} n_L = \dfrac{4aw}{4hw + 3L(h+w)} \\ n_w = \dfrac{4aL}{4hL + 3w(h+L)} \end{cases},$$

where *w*, *d* and *L* are the width, height, and length of the magnetic beam (*58*).

S3. Gripping force modelling of the microgripper

As shown in **Supplementary Fig. 6a**, the microgripper can be simplified as a two-segment structure linked by two picospring linkages. Assuming the picospring experiences a pure bending process in a constant curvature model, the elastic torque of each picospring is approximately given by [26]

$$T^{\mathrm{e}} = \frac{ab^2E}{2}\left(\frac{\Delta\varphi}{\varphi_0 - \Delta\varphi}\right) \quad \text{or} \quad \frac{1}{T^e} = \frac{2\varphi_0}{ab^2E}\frac{1}{\Delta\varphi} - \frac{2}{ab^2E},$$

E = the elastic modulus of the picospring;

a = the cross-section dimension perpendicular to the radial direction of the picospring;

b = the cross-section dimension parallel to the radial direction of the picospring;

φ_0 = the initial angle in radians of the arc picospring;

$\Delta\varphi$ = the angular deflection of the picospring in radians;

valid for b ≪ the curvature radius of the picospring.

When the microgripper is opened by the magnetic field, the magnetic torques on the magnetic rigid segments (fingers) are balanced by the elastic torques of the two arc picosprings, satisfying $T^e = \frac{T^m}{2}$. The angular deflection $\Delta\varphi$ is twice the rotation angle of the rigid finger $\Delta\theta$. The magnetic torque T^m is given by equation (2) as mentioned in section 2.1. We depict the measurement results for $\frac{1}{T^e}$ and $\frac{1}{\Delta\varphi}$ in **Supplementary Fig. 14b**. The elastic torque of the picospring T^e can be then approximatively determined from the angular deflection and the linear fitting curve of $\frac{1}{T^e}$ and $\frac{1}{\Delta\varphi}$.

When gripping an object, a cell for example, each finger of the microgripper is in a static equilibrium state satisfying $2T^e - T^m = Fl_1 \sin\theta$, where F represents the gripping force and l_1 is the distance from the gripping position to the hinge joint of the picospring. **Supplementary Fig.**

6c shows the gripping force with respect to the opening angle of the microgripper ($\Delta\varphi$). The gripping force on a HeLa cell under 4 mT shown in **Fig. 14d** is 2.4 pN with an average stress of 7.6 mPa, assuming full contact of the spheroidal cell membrane with a diameter of 10 μm. Higher gripping forces could be obtained with less compliant springs under higher magnetic fields.

S4. Dynamical model of the micropenguin locomotion based on shape morphing

By programming the time sequences of energy storing and releasing processes, the picosprings endow soft microrobots with complex motion modes under remote magnetic fields. As shown in **Fig. 5A**, the micropenguin motion has 4 phases. Phase 1: the micropenguin flippers are gradually closed by the magnetic torque at the high magnetic field, generating the 1st forward movement along *x*+ direction; Phase 2: the micropenguin is turned upwards and opens its flippers vertically by the elastic torque at the base field. The backward movement then occurs vertically as the 1st side displacement along *z*+ direction; Phase 3: the micropenguin with opened wings is turned back towards *x*+ and then close its wings to generate the 2nd forward movement; Phase 4: the micropenguin is turned downwards and opens its flippers to generate the 2nd side displacement along *z*- to counteract the 1st side displacement at *z-axis*. Then the micropenguin is turned horizontally back to its starting orientation and posture to start another cycle. In the fin-close process, the penguin is always kept horizontally to generate the forward movement. Meanwhile, it is periodically turned downwards and upwards to keep the vertical movement symmetry with 0 displacement vertically. Thus, the micropenguin's averaged velocity is defined as

$$v_{av} = \frac{2s}{2t_{close} + 2t_{open} + t_{r-close} + t_{r-open}}$$

, where s is the total forward displacement after the flippers close at one stroke, and t_{close}, t_{open}, $t_{r\text{-}close}$ and $t_{r\text{-}open}$ represent the flipper close, open, and two turning periods. Due to the time symmetry of $T_{close} = T_{open}$, we can deduce

$$\frac{1}{v_{av}} = \frac{2}{v_{close}} + \frac{1}{2s}\left(\frac{1}{4\omega_{r-close}} + \frac{1}{4\omega_{open}}\right)$$

, where v_{close} is the averaged velocity during the flipper close process, and $\omega_{r\text{-}close}$ *and* $\omega_{r\text{-}open}$ are the turning frequencies. This equation describes the basic principle of the micropenguin locomotion, also valuable in guiding the design of robots of other similar geometries. We know that v_{close} and s are positively relevant to the high magnetic field, when $\omega_{r\text{-}close}$ *and* $\omega_{r\text{-}open}$ are the highest rotating frequencies *i.e.* the step-out frequencies, which are given by $\omega_{so} = \frac{\mu}{c}\frac{n_l - n_r}{2n_l n_r} v B^2$ for the soft magnetic material (59). Accordingly, the average swimming velocity of the micropenguin is positively defined by the high field. The actuation strategy is therefore established as: setting the actuation field (high magnetic field) as high as possible; setting the rotating frequency as high as possible before the step-out frequency.

Modeling the flippers closing process helps to find the appropriate magnetic field and stroke time to develop the control strategy for the micropenguin and other possible microrobots in this locomotion mode The actuation source of the micropenguin is the magnetic torque on its two flippers. The magnetization of the magneto-elastomeric material can be found in **Supplementary Fig. 13**. Since the net displacement is donated by the stoke during the flippers closing, a dynamical model is devised to get a better understanding on this process. As shown in **Extended Data Fig. 3d**, the micropenguin is simplified as a three-segment structure connected by two picospring

linkages. The inertia is ignored here. Based on the Navier-Stokes equations at low-Reynolds-number condition, the micropenguin's motion satisfies $\begin{cases}\sum_{i=1}^{3}\mathbf{F}_i = 0\\ \sum_{i=1}^{3}\mathbf{T_i} = 0\end{cases}$, or $\begin{cases}\mathbf{F_1^h} + \mathbf{F_2^h} + \mathbf{F_3^h} = 0\\ \mathbf{T_i^h} + \mathbf{T_i^m} + \mathbf{T_i^e} = 0\end{cases}$ as a three-segment model connected by picospring linkages, where $\mathbf{F_i^h}$, $\mathbf{T_i^h}$, $\mathbf{T_i^m}$ and $\mathbf{T_i^{\mathit{e}}}$ represent hydrodynamic force, the hydrodynamic torque, the magnetic torque and the elastic torque of each segment, respectively (*60*). Each segment is regarded as slender and thus the hydrodynamic drag parallel and perpendicular to the long axis of the segment satisfies $\begin{cases}F_i^{\parallel} = \xi_i^{\parallel} l_i v_i^{\parallel}\\ F_i^{\perp} = \xi_i^{\perp} l_i v_i^{\perp}\end{cases}$, where $v_i^{\parallel}$ $(resp.\, v_i^{\perp})$ and $\xi_i^{\parallel}$ $(resp.\ \xi_i^{\perp})$ represent the projection of the segment velocity on the direction parallel ($resp.$ perpendicular) to the axial axis of the segment and the related drag coefficient. These parameters can be calculated by

$$\begin{cases}\xi_i^{\parallel} = \dfrac{2\pi\eta}{\ln\left(2L_i/w_i\right)},\\ \quad \xi_i^{\perp} = 2\xi_i^{\parallel}\end{cases}$$

where η is the environment viscosity, L and w are the length and width of the segment (*31*). The elastic torque of the arc model is based on the lateral bending of the original coil spring, which can be simplified as a linear process of an elastic rod (*61*) here as

$$\begin{cases}T^e = k^e\,\alpha\\ \alpha = \dfrac{1}{2}(\theta_0 - \theta)\end{cases},$$

where k^e and α represents the bending stiffness and angle, when θ and θ_0 are the instantaneous and initial orientation angles of the flipper. The distance between the endpoints of the spring is given by the chord of the bent arc as $L_c = \frac{L_s}{\alpha} sin\alpha$, assuming no length change on the arc length L_s during a linear bending.

At low Reynolds number, the rotation of the flipper L_1 satisfies

$$T_1^m - T_1^h - T_1^e = 0, \qquad (2).$$

During the stroke when the flippers close, the spring is bent, giving the elastic torque as

$$T_1^e = k^e\,\alpha\,, \qquad (3).$$

The actuation comes from the magnetic torque applied on the two flippers, each flipper can be regarded as a magnetic beam with a magnetic torque satisfying Equation (2).

The hydrodynamic torque of the flipper L_1 is obtained as

$$T_1^h = \frac{1}{2}\xi_1^{\perp} v_1^{\perp} L_1^2. \quad (4)$$

Its movement can be decomposed as its rotation and the translational movement of the whole robot, giving

$$\begin{cases} v_1^{\parallel} = v_1^x \cos\theta - v_1^y \sin\theta\,, v_1^{\perp} = v_1^x \sin\theta - v_1^y \cos\theta\\ v_1^x = \dot{x} - \dfrac{\dot{\theta}}{2}(L_1 sin\theta + L_c sin\alpha), \quad v_1^y = -\dfrac{\dot{\theta}}{2}(L_1 cos\theta + L_c cos\alpha)\end{cases},$$

where $\dot{\theta}$ is the angular speed of the fin, and $\dot{x} = v_3^{\parallel}$ is the locomotion velocity of the torso.

When the flippers rotate symmetrically, the axial hydrodynamic forces of the segments can be written as

$$\begin{cases} F_1^h = F_2^h = \frac{1}{2}F_3^h \\ F_1^h = \xi_1^{\parallel} v_1^{\parallel} L_1 cos\theta + \xi_1^{\perp} v_1^{\perp} L_1 sin\theta \, ' \\ F_3^h = \xi_3^{\parallel} \dot{x} L_3 \end{cases} \quad (5).$$

Simultaneous equations of the torque balance (2) and the force balance (5) comprised of (1), (3) and (4) can then be analyzed numerically by using a Runge-Kutta 4th order iterative method by Matlab.

The boundary conditions and essential parameters can be obtained by the robot geometry as shown in Table 1. The deflection angle of the spring linkage relevant to the magnetic field is characterized by experiments and depicted in **Supplementary Fig. 15a**. The deflection angle is obtained as half of the rotation angle of the flipper. The bending stiffness of the spring is calculated as the slope by fitting the deflection angle and the magnetic (elastic) torque deduced by Equation (2). The bending curve of the coil picospring shows high linearity under small deflection indicating a pure bending process of the picospring. **Supplementary Fig. 15b** depicts the maximum (step-out) frequency of the micropenguin rotation relevant to the magnetic field. The positive relation of them indicate a higher magnetic field for an optimal actuation strategy on account of the rotation process.

During modelling, the step size of the time, angle and magnetic field was set as 0.001 s, 0.001 deg and 0.01 mT, respectively. **Supplementary Fig. 15c-f** show the result from the sweeping process of the magnetic fields. Theoretical results show that the micropenguin achieves larger total displacement and average velocity under the actuation of a stronger magnetic field in one stroke. Nevertheless, the increasing of the displacement levels off when over ~10 mT (**Supplementary Fig. 15c** and **5d**). Under the simultaneous control of the magnetic torque and elastic force, the time to reach a full deformation increases rapidly under the magnetic field below 8.7 mT then decreases. In addition, the average velocity, the maximum deflection angle and the maximum displacement are all positively relative to the field strength, indicating the choice of a high magnetic field. However, the increasing of displacement obviously slows down under the field over 15 mT. Thus, 16 mT are used as the high field for actuation out of considerations of the actuation efficiency and the cooling of the electromagnetic coils. The deflection angle by calculation is slightly higher than measurement. This can be attributed to the nonlinearity of the spring deformation and the flipper magnetization of the real robot. **Supplementary Fig. 15g** and **h** show the instantaneous bending and velocity of the micropenguin actuated at 16 mT. Under a certain magnetic field, the micropenguin's forward velocity decreases over time during one stroke. The micropenguin accomplishes over 75% of its displacement in the first 1 s while the whole process needs almost 4 s. The stroke time is thus set as 1 s, at which the forward velocity has largely decreased, for example by more than 70% under a magnetic actuation field of 16 mT (**Supplementary Fig. 15g**). Notably, our quasi-static analysis shows that the velocity curve reaches its stabilized state within the first 0.005 s (inset of **Supplementary Fig. 15h**), which is highly consistent with the locomotion behaviour at low Reynolds number. Based on these modelling result, the stroke period is set as 1 s and the whole cycle is set as 9 s with a high magnetic field of 16 mT in our initial control strategy. The measured velocity during one stroke is ca. 1.79 μm/s, higher than the modelling value (1.36 μm/s). The geometry error brought by the model simplification donates this variation.

Combing the flipper flapping and the rotation of the whole body, the micropenguin is actuated in an orientation-switching strategy. **Extended Data Fig. 6a.** illustrates the time sequence of the magnetic field of one control cycle in this strategy. As illustrated in **Fig. 6A**, the micropenguin first closes its flippers by aligning them towards the field direction of the magnetic actuation field, deforming the elastic linkages and achieving forward velocity (+*x*) by pushing the fluid backward (Phase 1-2 or 3-4). The micropenguin is then rotated by 90°. After that, it opens its flippers along the vertical directions (+*z* or -*z* in sequence, Phase 2-3 and 4-1) avoiding any -*x* displacement. The symmetric displacements along +*z* and -*z* directions during the flippers opening processes ensure no *z-axis* drifting of the micropenguin locomotion. In order to save time for the rotation we use the maximum rotation speed for the magnetic field defined by the step-out frequency[27]. The micropenguin was continuously actuated by periodically repeating the above movement cycle under the cycled time-sequential magnet field (see the locomotion sequence in **Fig. 6B**). Although the forward velocity and the step-out frequency both theoretically increase with the magnetic field, experimental results show no significant increase in the average velocity when the magnetic actuation field is over 20 mT (**Extended Data Fig. 3c**). The nonlinear magnetization of the material and the cut-off of the stroke time during the experiment minimize the influence of the increasing magnetic field over 20 mT. The locomotion velocity can be largely increased by overlapping the micropenguin rotation process with the flippers open/close process and reducing the cycle length. The micropenguin's velocity increases over 3 times under a more efficient manner with a cycling time of 5.5 s as shown in **Extended Data Fig. 3b**.

S5. Dynamical model of the microturtle locomotion based on shape morphing

The modeling of the microturtle is basically the same with the micropenguin except for two points: (1) The spring legs connecting the flippers onto the torso cannot be ignored, so the dynamical equations of the flippers need to include the elastic mechanics of the spring legs; (2) Unlike the micropenguin which only moves along one direction in one stroke, the microturtle's stroke includes both translation and rotation at *x*-y plane. Thus, the dynamical equations of the movement of the flippers as well as the torso should be constructed with generalized coordinates as

$$G = \begin{pmatrix} x \\ y \\ \theta \end{pmatrix}, \dot{G} = \begin{pmatrix} \dot{x} \\ \dot{y} \\ \dot{\theta} \end{pmatrix}.$$

The theoretical model of the microturtle then can be established by the same equation set. However, deriving a comprehensive model for such complex gait goes beyond the scope of the present study. Solving such equations would be difficult when the geometry cannot be properly simplified considering the noneligible influence of the geometry details. For example, the large width of the torso hinders the microturtle locomotion. Narrowing the torso can raise the velocity, nevertheless make the swimming more unsteadily. Therefore, a FEA analysis is finally implemented.

Owing to the multiple-exposure fabrication strategy as shown in **Supplementary Fig. 16**, the microturtle owns both magnetic and nonmagnetic components, enabling variable and complex movement gaits under different magnetic fields. We implement a sequential-motion control strategy, of which the time-sequential magnetic field in one cycle is shown in **Extended Data Fig. 6b.** As shown in Fig. 6C, the microturtle is first turned by 15° from its initial direction along +*y* to

generate a preserved angle by the magnetic base field (0-1 s). Then the base field is applied perpendicular to the long axis of the microturtle, slightly opening the flippers (1-1.5 s). From 1.5 to 2.5 s, the flippers are widely opened and aligned towards the magnetic actuation field, pushing the microturtle forward with rotation due to the asymmetric rotation of the right and left flippers. Here, the torso rotation lags behind the flippers rotation due to the buffering of the elastic hinges, superior to rigid hinges. From 2.5 to 3 s, the flippers rotate back toward the magnetic field under the buffering by relaxing springs. Consequently, sequential movements of the right flippers, torso and left flippers are generated in one cycling period (0-3 s). The locomotion velocity shows a positive relationship with respect to the magnetic field (**Extended Data Fig. 6c**). Small-scale soft robots generally face a persistent challenge in achieving sufficient locomotion efficiency. While their high flexibility allows for accommodating large deformations and sensing environmental forces without permanent damage, their actuation forces are indeed restricted by the deformation and recovery rates. Improved output power and more complex movement modes could be attainable by combing the picosprings with, for example, latch systems[68] or gear-based amplifier system[69].

Despite different actuation mechanisms, the steering of all the present microrobots can be done by simply changing the magnetic field direction (**Supplementary Fig. 17**). Nonetheless, two points need to be noticed: (1) Since the magnetic alignment has no selectivity in the two directions of the easy axis in the magnetic structure, the steering angle should be set below 90°; (2) The steering speed should be lower than the step-out frequency of the robot. The control strategies of the whole locomotion process including steering can be programmed in one step, fully open to future autonomous control based on microscopic maps or real-time image recognition.

References:

1. Miskin, M. Z. *et al.* Electronically integrated, mass-manufactured, microscopic robots. *Nature* **584**, 557–561 (2020).
2. Aghakhani, A., Yasa, O., Wrede, P. & Sitti, M. Acoustically powered surface-slipping mobile microrobots. *Proc. Natl. Acad. Sci. U. S. A.* **117**, 3469–3477 (2020).
3. Dong, X. *et al.* Toward a living soft microrobot through optogenetic locomotion control of Caenorhabditis elegans. *Sci. Robot.* **6**, (2021).
4. Magdanz, V. *et al.* IRONSperm: Sperm-templated soft magnetic microrobots. *Sci. Adv.* **6**, (2020).
5. Xin, C. *et al.* Environmentally adaptive shape-morphing microrobots for localized cancer cell treatment. *ACS Nano* **15**, 18048–18059 (2021).
6. Wang, Y., Nitta, T., Hiratsuka, Y. & Morishima, K. In situ integrated microrobots driven by artificial muscles built from biomolecular motors. *Sci. Robot.* **7**, (2022).

7. Yasa, I. C., Ceylan, H., Bozuyuk, U., Wild, A. M. & Sitti, M. Elucidating the interaction dynamics between microswimmer body and immune system for medical microrobots. *Sci. Robot.* **5**, (2020).

8. Maeda, S., Hara, Y., Sakai, T., Yoshida, R. & Hashimoto, S. Self-Walking Gel. *Adv. Mater.* **19**, 3480–3484 (2007).

9. Li, J. *et al.* Development of a magnetic microrobot for carrying and delivering targeted cells. *Sci. Robot.* **3**, eaat8829 (2018).

10. Ghosh, S. & Ghosh, A. Mobile nanotweezers for active colloidal manipulation. *Sci. Robot.* **3**, (2018).

11. Palagi, S. *et al.* Structured light enables biomimetic swimming and versatile locomotion of photoresponsive soft microrobots. *Nat. Mater.* **15**, 647–653 (2016).

12. Li, H., Go, G., Ko, S. Y., Park, J.-O. & Park, S. Magnetic actuated pH-responsive hydrogel-based soft micro-robot for targeted drug delivery. *Smart Mater. Struct.* **25**, 27001 (2016).

13. Pak, O. S., Gao, W., Wang, J. & Lauga, E. High-speed propulsion of flexible nanowire motors: Theory and experiments. *Soft Matter* **7**, 8169–8181 (2011).

14. Zeng, H. *et al.* Light-fueled microscopic walkers. *Adv. Mater.* **27**, 3883–3887 (2015).

15. Rubin, S., Young, M. H. Y., Wright, J. C., Whitaker, D. L. & Ahn, A. N. Exceptional running and turning performance in a mite. *J. Exp. Biol.* **219**, (2016).

16. Boryshpolets, S., Pérez-Cerezales, S. & Eisenbach, M. Behavioral mechanism of human sperm in thermotaxis: A role for hyperactivation. *Hum. Reprod.* **30**, (2015).

17. Shunmugam, A. P., Subramanian, G. & Fernandez, J. G. Measurements of the swimming speeds of motile microorganisms using object tracking and their correlation with water pollution and rheology levels. *Sci. Rep.* **11**, (2021).

18. Travnickova, J. *et al.* Macrophage morphological plasticity and migration is Rac signalling and MMP9 dependant. *Sci. Rep.* **11**, (2021).

19. Cheong, F. C. *et al.* Rapid, High-Throughput Tracking of Bacterial Motility in 3D via Phase-Contrast Holographic Video Microscopy. *Biophys. J.* **108**, (2015).

20. Liao, K. H., Qian, Y. & MacOsko, C. W. Ultralow percolation graphene/polyurethane acrylate nanocomposites. *Polymer (Guildf).* **53**, 3756–3761 (2012).

21. Cui, J. *et al.* Nanomagnetic encoding of shape-morphing micromachines. *Nature* **575**, 164–168 (2019).

22. Hu, X. *et al.* Magnetic soft micromachines made of linked microactuator networks. *Sci. Adv.* **7**, eabe8436 (2021).

23. Dreyfus, R. *et al.* Microscopic artificial swimmers. *Nature* **437**, 862–865 (2005).

24. Qiu, T. *et al.* Swimming by reciprocal motion at low Reynolds number. *Nat. Commun.* **5**,

5119 (2014).

25. Kim, Y., Parada, G. A., Liu, S. & Zhao, X. Ferromagnetic soft continuum robots. *Sci. Robot.* **4**, eaax7329 (2019).

26. Hibbeler, R. C. *Mechanics of Materials*. (Pearson Prentice Hall).

27. Mahoney, A. W., Nelson, N. D., Peyer, K. E., Nelson, B. J. & Abbott, J. J. Behavior of rotating magnetic microrobots above the step-out frequency with application to control of multi-microrobot systems. *Appl. Phys. Lett.* **104**, 144101 (2014).
